# Dietary indoles influence the AHR-ROR**γ**t axis and mucosal immune homeostasis in ART-treated SIV infection

**DOI:** 10.1172/jci.insight.201258

**Published:** 2026-04-02

**Authors:** Siva Thirugnanam, Alison R. Van Zandt, Alexandra B. McNally, Victoria A. Hart, Isabelle Berthelot, Cecily C. Midkiff, Lara A. Doyle-Meyers, David A. Welsh, Robert V. Blair, Andrew G. MacLean, Namita Rout

**Affiliations:** 1 Tulane National Biomedical Research Center, Covington, Louisiana, USA.; 2 Department of Microbiology and Immunology, Tulane University School of Medicine, New Orleans, Louisiana, USA.; 3 Department of Microbiology, Immunology and Parasitology, Louisiana State University School of Medicine, New Orleans, Louisiana, USA.; 4 Louisiana Cancer Research Center, New Orleans, Louisiana, USA.; 5 Tulane Center for Aging, Tulane University School of Medicine, New Orleans, Louisiana, USA.

**Keywords:** AIDS/HIV, Immunology, Inflammation, Cellular immune response, Innate immunity

## Abstract

HIV infection rapidly impairs the gastrointestinal barrier, contributing to persistent mucosal immune dysfunction, microbial translocation, and systemic inflammation despite antiretroviral therapy (ART). Using SIV-infected rhesus macaques on long-term ART, we investigated mechanisms underlying impairment in gut barrier–protective IL-17/IL-22 responses and the potential modulation of this pathway by dietary indoles. Longitudinal profiling of colonic epithelial and lamina propria cells revealed a selective loss of IL-17/IL-22–producing γδ T cells and type 3 innate lymphoid cells (ILC3s). This loss correlated with reduced expression of the transcription factors AHR and RORγt and was associated with elevated plasma markers of intestinal epithelial barrier disruption (IEBD), including intestinal fatty acid–binding protein (iFABP), zonulin, and LPS-binding protein (LBP). Targeting this transcriptional deficiency, dietary indole supplementation for 1 month restored colonic AHR^+^ IL-22–producing γδ T cells, RORγt^+^ ILC3s, and Vδ1 T cells, and was associated with reduced iFABP and zonulin levels. Immunohistochemical analyses further demonstrated enrichment of AHR/RORγt-coexpressing cells in the colon of indole-supplemented animals during chronic SIV infection on ART. Collectively, these findings indicate that disruption of the AHR-RORγt axis is a key pathogenic mechanism underlying persistent IEBD in chronic SIV/HIV infection. Modulation of AHR and RORγt signaling pathways in the gut may therefore represent a promising therapeutic strategy to reinforce mucosal barrier function and mitigate chronic inflammation in people living with HIV.

## Abstract 2

Our research shows that dietary indoles may help repair gut damage and reduce inflammation in chronic HIV by boosting key protective immune cells.

## Introduction

Chronic HIV/SIV infection is characterized by persistent immune activation and systemic inflammation, even with the successful suppression of viral replication through antiretroviral therapy (ART) (1, 2). A key driver of this pathology is the damage to the gastrointestinal tract, leading to a phenomenon known as “leaky gut” (3). This intestinal barrier dysfunction allows microbial products to translocate from the gut lumen into the bloodstream, fueling the chronic inflammation that contributes to incomplete immune recovery (3) and non-AIDS comorbidities including cardiovascular, kidney, and liver diseases (4). While ART is highly effective at controlling viremia and reducing some inflammatory markers, it often fails to fully restore the gut barrier and immune homeostasis (5, 6), highlighting the need for novel therapeutic strategies that specifically target the mechanisms of chronic inflammation and intestinal dysfunction.

HIV and SIV preferentially infect intestinal CD4^+^ Th17 cells, whose loss is linked to intestinal epithelial barrier disruption (IEBD), microbial translocation, systemic inflammation, and disease progression (5, 7). Chronic HIV also depletes protective Vδ2 γδ T cells while expanding inflammatory Vδ1 subsets, contributing to barrier damage (8). Vδ2 cells transiently expand and produce IL-17/IL-22 during acute SIV, then decline, inverting the Vδ2/Vδ1 ratio (9), suggesting a short-lived compensatory role. Elite and viral controllers maintain higher Vδ2 frequencies and IL-17 production than untreated or ART-treated people with HIV (10, 11). Group 3 innate lymphoid cells (ILC3s) similarly support barrier integrity (12, 13) but decline during long-term ART (13, 14), correlating inversely with plasma intestinal fatty acid–binding protein (iFABP). The mechanisms underlying functional loss of type 3 lymphocytes (Th17, γδ T, ILC3) in chronic treated infection, and whether they can be modulated to restore barrier function, remain unclear.

The functional integrity of type 3 effector lymphocytes is dependent on key transcription factors, such as T-box protein expressed in T cells (T-bet), which drives Th1 cell polarization, and transcription of retinoic acid–related orphan receptor γ isoform t (RORγt), which is essential for ILC3, γδ T, and Th17 cell development and function (15–17). The aryl hydrocarbon receptor (AHR), a ligand-activated transcription factor, also plays a critical role in regulating mucosal immunity and barrier function (16). AHR binding is a major inducer of transcription of RORγt, and activating AHR signaling via diet- or microbiota-derived ligands was shown to restore intestinal barrier integrity and function via IL-22 and IL-17 secretion, strengthening of tight junctions, and induction of IL-10R (18).

Despite their critical roles, the dynamics of type 3 effector lymphocytes during acute and chronic SIV infection, and their response to long-term ART, remain incompletely characterized. Here, we define the compartmentalized transcriptional and functional profiles of circulating and colon-resident γδ T cells and ILC3s across the course of SIV infection and ART in rhesus macaques. We further evaluate the effects of dietary AHR ligand supplementation on gut barrier integrity and mucosal immune function. Our findings highlight a potential role for the AHR–RORγt–IL-22 axis in supporting gut barrier integrity and limiting chronic inflammation in ART-treated HIV/SIV infection.

## Results

### iFABP recovery during ART parallels decline in SIV-induced proinflammatory cytokines.

**Figure 1:**
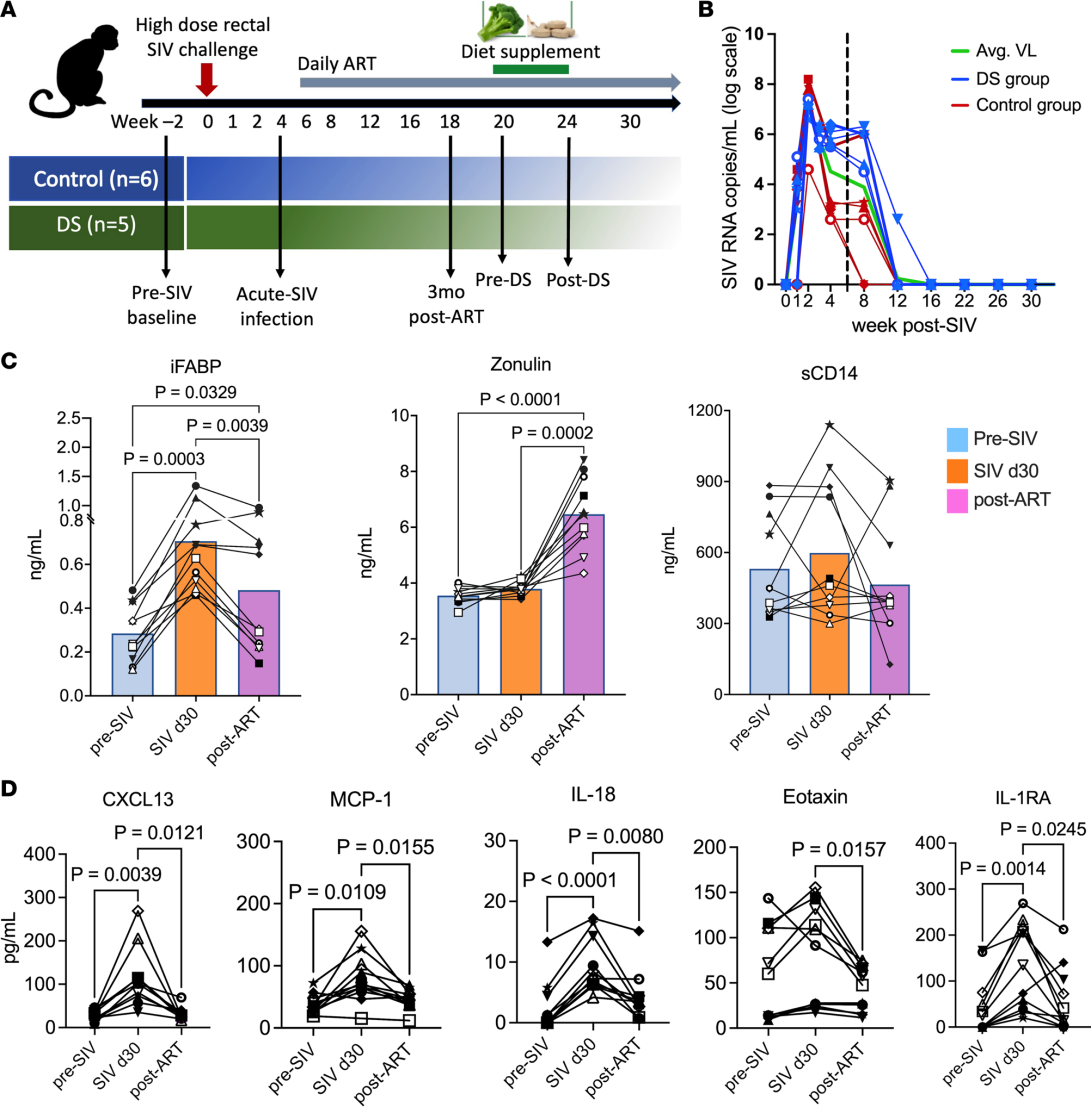
Plasma biomarkers of epithelial barrier disruption, microbial translocation, and inflammatory cytokines during SIV infection and ART. (**A**) Study design. SIV-infected rhesus macaques (*n* = 11) received daily ART (TDF, FTC, DTG). Baseline blood and gut biopsy samples were collected at week –2 and day 0 SIV challenge, and ART was initiated at 6 weeks after SIV infection. (**B**) Plasma SIV RNA levels over 30 weeks. Dashed line marks ART initiation, blue symbols represent animals in the DS group, red symbols represent control group, and green line shows average viral loads (VL) for all animals. (**C**) Plasma iFABP, zonulin, and sCD14 concentrations at pre-SIV baseline, day 30 after SIV infection (acute SIV), and 12 weeks after SIV+ART (post-ART). (**D**) Plasma CXCL13, MCP-1, IL-18, eotaxin, and IL-1RA levels at pre-SIV baseline, acute SIV (day 30), and 12 weeks post-ART time points. Filled symbols represent control group, and open symbols represent DS group animals. Data are shown as mean ± SEM and *P* values using repeated-measures 1-way ANOVA.

**Figure 2:**
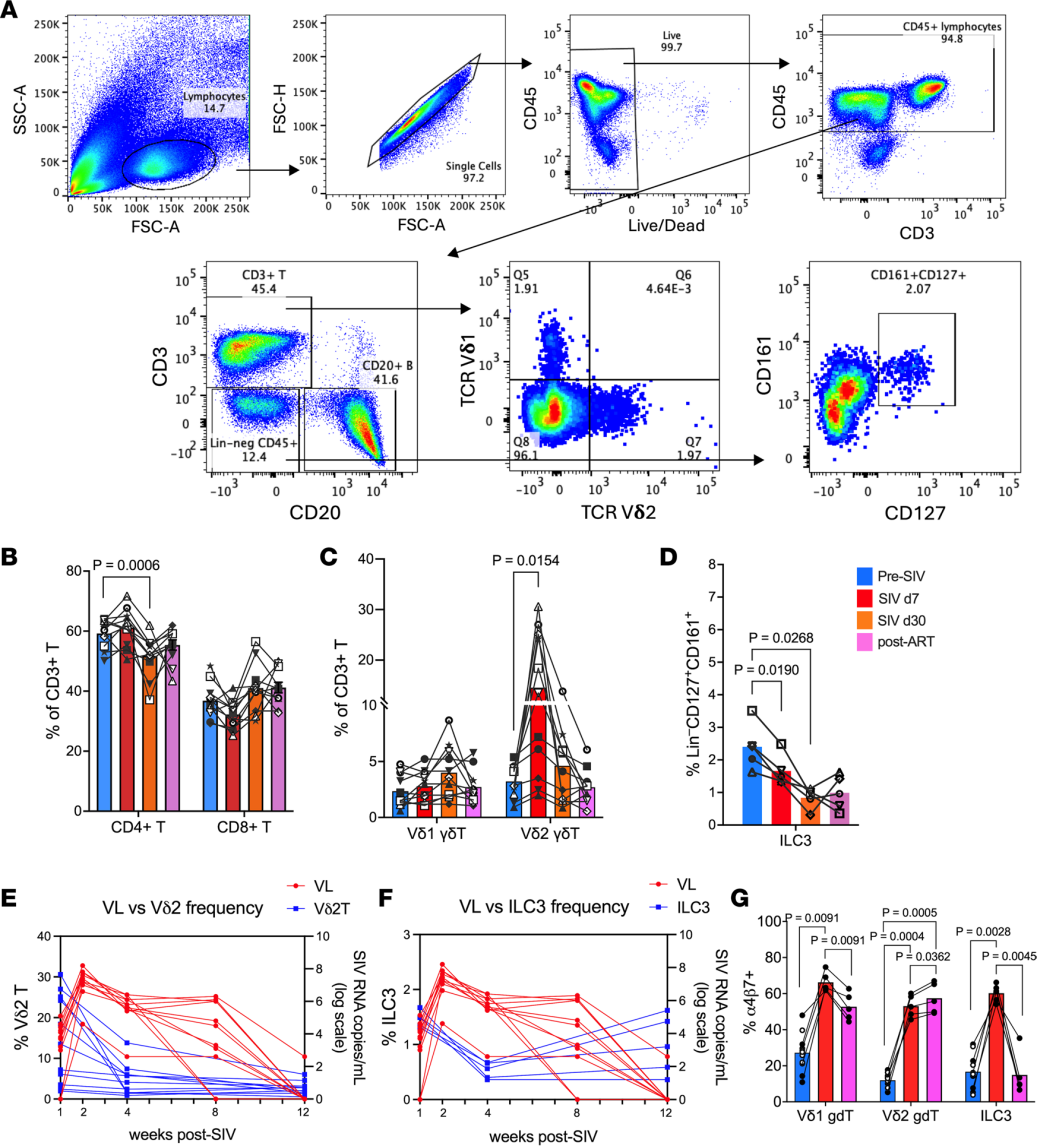
Temporal dynamics of Vδ2 T cell and ILC3 depletion during acute SIV infection and their relationship to peak viremia. (**A**) Representative gating schematic for Vδ1 T cells, Vδ2 T cells, and ILC3s in PBMCs. (**B**–**D**) Frequencies of CD4^+^ and CD8^+^ T cells (**B**), Vδ1 and Vδ2 γδ T cells (**C**), and ILC3s (**D**) in peripheral blood of study animals at baseline (2 weeks before SIV infection), acute SIV infection (day 7 and day 30 after SIV), and 12 weeks after SIV+ART (post-ART). Filled symbols represent control group, and open symbols represent DS group animals. (**E** and **F**) Circulating immune cell frequencies (blue, left *y* axis) and SIV viral load in log scale (red, right *y* axis) at each sampling time point for Vδ2 T cells (**E**) and ILC3s (**F**). (**G**) Frequencies of PBMC Vδ1 T cells, Vδ2 T cells, and ILC3s expressing integrin α_4_β_7_ at indicated time points. Data are shown as mean ± SEM by paired ANOVA with mixed-effects analysis using Tukey’s test for integrin α_4_β_7_.

Two cohorts of rhesus macaques were infected with SIV and, following peak viremia, were subsequently treated with daily ART and followed for more than 6 months, with one group of 5 macaques receiving broccoli-based dietary AHR ligands to target gut inflammation (Figure 1A). Data points represent a combination of animals from a previously described cohort (9) and the current dietary intervention cohort to demonstrate model concordance. To ensure a robust baseline, we first confirmed the virological and immunological concordance between our current and previous control cohorts (see Methods), allowing for pooled analysis in Figures 1 and 2. Plasma viremia peaked at 2 weeks (7.16 log_10_ copies/mL) and ranged between 3 and 6.4 log_10_ copies/mL at 4 weeks (Figure 1B). Daily ART (tenofovir disoproxil fumarate [TDF], emtricitabine [FTC], and dolutegravir [DTG]) began at 6 weeks after infection, achieving viral suppression below the assay limit of detection within 6 weeks. Plasma inflammatory and IEBD biomarkers were measured at baseline (pre-SIV), acute infection (day 30 after SIV), early ART (3 months after SIV+ART), and late chronic ART phases (>5 months of ART).

iFABP, a marker of enterocyte damage, rose significantly during acute infection (4 weeks, *P* = 0.019) and remained elevated despite ART, whereas zonulin increased later, 3 months after ART (*P* = 0.015), suggesting that early epithelial injury triggers tight junction disruption (Figure 1C). Soluble CD14 (sCD14), a marker of microbial translocation, remained unchanged. Circulating proinflammatory cytokines (CXCL13, IL-18, eotaxin, IL-1RA) surged at 4 weeks after SIV and partially declined with ART (Figure 1D). These findings indicate that SIV-induced IEBD occurs early, with epithelial damage leading to zonulin-mediated tight junction disruption and inflammation, and although ART partially normalizes iFABP and reduces inflammation, zonulin remains elevated beyond baseline, likely contributing to persistent microbial translocation and inflammation despite effective viral suppression.

### Depletion of circulating Vδ2 T cells and ILC3s during acute SIV infection is associated with peak viremia and elevated integrin α_4_β_7_ expression.

Extensive HIV/SIV replication in gut-associated lymphoid tissue depletes CCR5^+^ and Th17 CD4^+^ T cells, disrupting the epithelial barrier and promoting microbial translocation. Compensatory mucosal populations, including γδ T cells and ILC3s, support barrier integrity via IL-17 and IL-22 production (9, 13). We longitudinally tracked CD3^+^Vδ1^+^ T cells, CD3^+^Vδ2^+^ T cells, and lineage^–^CD8α^–^CD127^+^CD161^+^ ILC3 populations during acute SIV infection and subsequent ART. Despite differences in CD4 expression, Vδ1 and Vδ2 T cells both expressed high CD95, consistent with a memory/activated phenotype (Supplemental Figure 1; supplemental material available online with this article; https://doi.org/10.1172/jci.insight.201258DS1), and showed similar IL-17 and IL-22 production at steady state. A trend toward increased CD8^+^ T cell frequencies was observed at day 30 in response to acute SIV infection, while the CD4^+^ T cell compartment exhibited a significant decline following peak viremia and returned to baseline at 3 months after ART (Figure 2B). Vδ2 γδ T cells underwent a rapid, transient expansion during early acute infection (day 7) before returning to baseline levels by day 30 (Figure 2C), corroborating our earlier findings (9). This transient expansion reflects an acute proliferative response, which was followed by a significant contraction that coincided with peak viral replication. On the other hand, a rapid and progressive decline in ILC3s beginning during peak viremia was observed (Figure 2D). Despite their divergent kinetics at 1 week after infection, the decline of both peripheral Vδ2 T cells and ILC3s between weeks 1 and 4 was associated with viral dynamics, displaying an inverse relationship with plasma viral loads (Figure 2, E and F). Notably, the depletion of these circulating populations was accompanied by a significant upregulation of the gut-homing integrin α_4_β_7_ on Vδ1, Vδ2, and ILC3 subsets (Figure 2G). These data suggest that the loss of these critical innate-like populations from the circulation during peak viremia is likely driven by enhanced trafficking to the inflamed intestinal mucosa.

### SIV-driven Th1 polarization persists in circulating γδ T cells and ILC3s during ART, marked by T-bet upregulation and RORγt/AHR downregulation.

**Figure 3:**
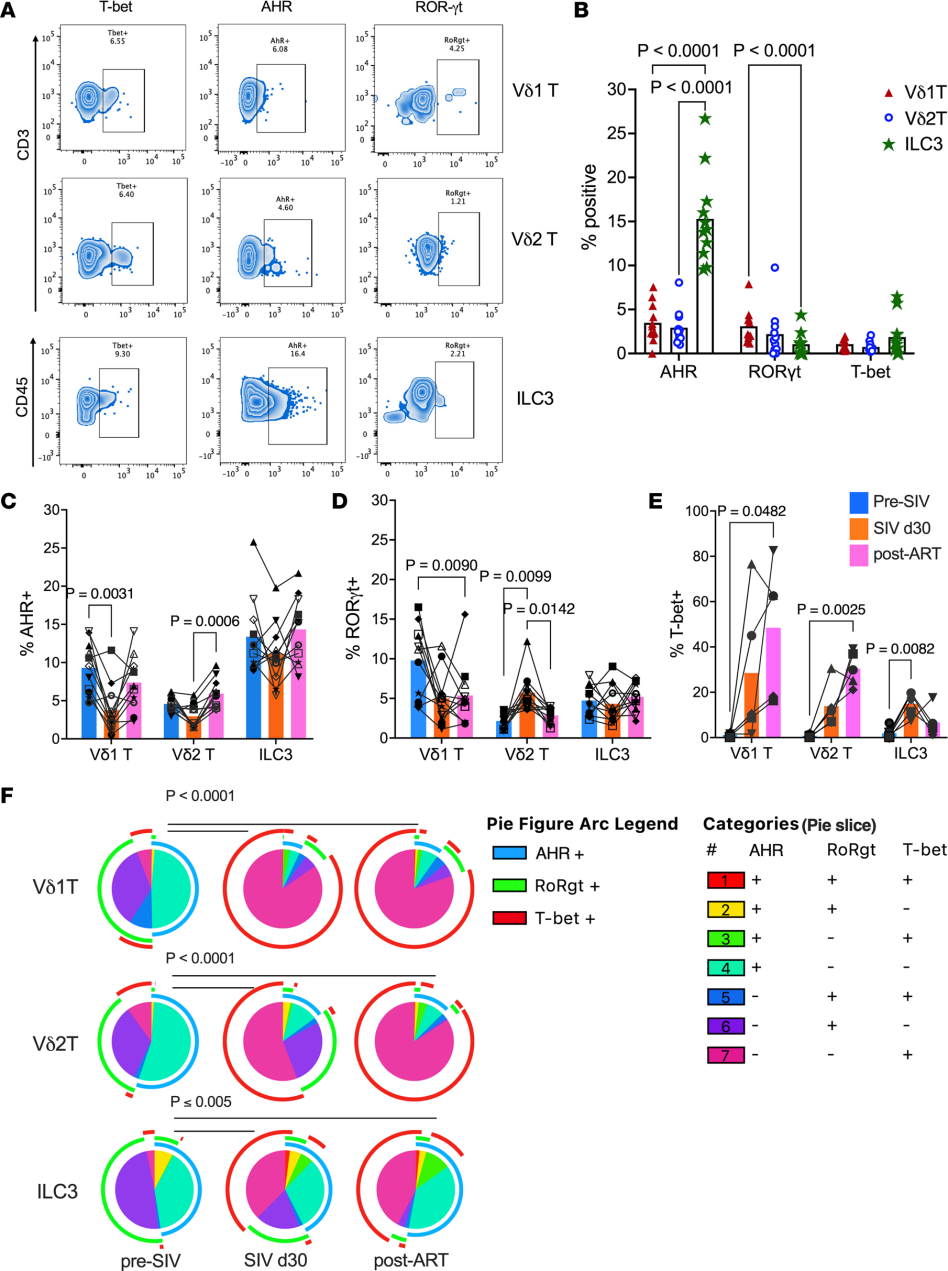
Transcription factor expression in circulating type 3 immune cells during SIV infection and ART. (**A**) Representative flow cytometry plots showing expression of the transcription factors AHR, RORγt, and T-bet in Vδ1 T cells, Vδ2 T cells, and ILC3s. (**B**) Frequencies of AHR, RORγt, and T-bet expression in PBMC Vδ1 T, Vδ2 T, and ILC3 subsets at baseline. (**C**–**E**) Longitudinal changes in the frequencies of AHR^+^ (**C**), RORγt^+^ (**D**), and T-bet^+^ (**E**) Vδ1 T, Vδ2 T, and ILC3 populations evaluated before SIV, at acute SIV infection (day 30 after SIV), and 12 weeks after SIV+ART (post-ART). Data are shown as mean ± SEM and paired ANOVA test *P* values. Filled symbols represent control group, and open symbols represent DS group animals. (**F**) Pie charts depicting the relative expression of various combinations of AHR, RORγt, and T-bet by Vδ1 T, Vδ2 T, and ILC3 populations from pre-SIV baseline through SIV infection and post-ART. Pie arcs represent expression of individual transcription factors, and pie slices represent the proportion of coexpressed transcription factors under each category. Significant differences between each time point for the overall phenotype were evaluated by SPICE permutation test.

The shift in Th1/Th17 balance in mucosal tissues is a well-documented hallmark of HIV/SIV infections (19, 20). To examine changes in functional immunophenotype of circulating ILC3s and γδ T cells, changes in their transcription factor profiles were evaluated at baseline (Figure 3, A and B), following SIV infection, and during the immune reconstitution phase of ART (Figure 3, C–E). At baseline, γδ T cells and ILC3s exhibited higher frequencies of cells expressing the Th17-associated transcription factors AHR and RORγt compared with those expressing the Th1-associated factor T-bet (Figure 3B). Notably, among the 3 subpopulations, ILC3s displayed significantly higher RORγt mean fluorescence intensity (MFI) relative to Vδ1 T cells and lower T-bet MFI in comparison with both Vδ1 and Vδ2 T cells, suggesting a predominant Th17-type functional phenotype (Supplemental Figure 2).

Following SIV infection, AHR^+^ Vδ1 T cells transiently declined but normalized with ART (Figure 3C). Vδ2 T cells, by contrast, showed no significant change in AHR expression during acute infection but exhibited increased AHR expression during ART, indicating enhanced AHR signaling in the setting of viral control. Notably, Vδ2 T cells also showed an early increase in RORγt expression after infection, which returned to baseline after 2 months on ART, coinciding with an upregulation of T-bet (Figure 3, D and E). These findings suggest that ART restores the frequency of certain Th17-associated transcriptional profiles, such as AHR^+^ Vδ2 T cells and RORγt^+^ Vδ1 T cells, but may also promote a shift in type 3 immune cells from a Th17-like phenotype (AHR^+^RORγt^+^) toward a Th1-like phenotype (T-bet^+^) during acute infection and viral suppression (Figure 3F). This dynamic may reflect immune reprogramming associated with treated SIV infection, where ART partially restores immune homeostasis but may also skew innate-like T cell subsets toward Th1-type responses.

### Compartmentalized γδ T cell and ILC3 dynamics in colonic epithelium versus lamina propria during SIV and ART.

**Figure 4:**
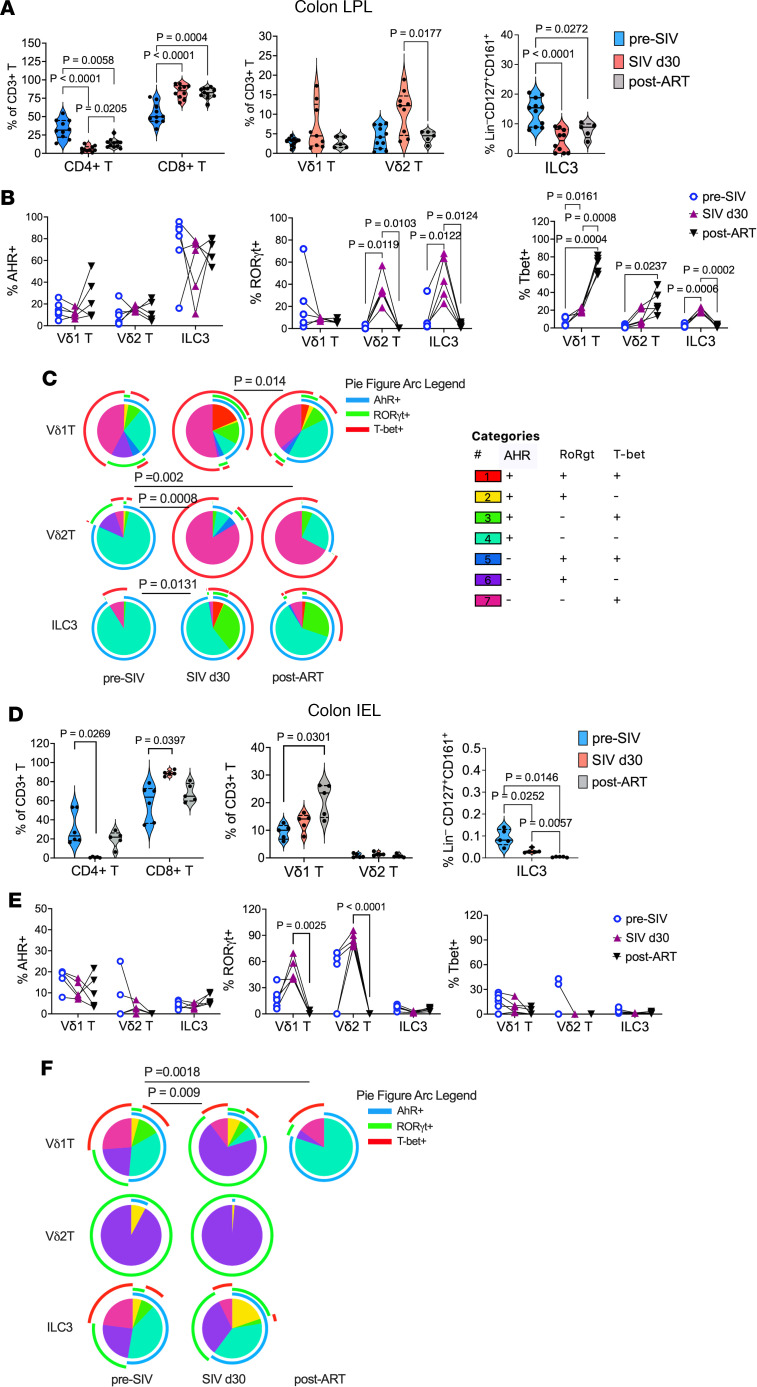
Frequencies of colonic mucosal γδ T cells and ILC3s and expression of Th1/Th17-associated transcription factors during acute SIV infection and ART. (**A**) Longitudinal frequencies of CD4^+^ T, CD8^+^ T, Vδ1 T, Vδ2 T, and ILC3 populations in the LPLs from colon biopsies at indicated time points using the gating strategy described in Figure 2A. (**B**) AHR, RORγt, and T-bet expression by colonic LPL Vδ1 T, Vδ2 T, and ILC3 populations. (**C**) Pie charts depicting the relative expression of AHR, RORγt, and T-bet by Vδ1 T, Vδ2 T, and ILC3 populations in colonic LPLs isolated from pre-SIV baseline through SIV infection and ART. (**D**) Longitudinal frequencies of CD4^+^ T, CD8^+^ T, Vδ1 T, Vδ2 T, and ILC3 populations in colonic IELs at indicated time points. (**E**) AHR, RORγt, and T-bet expression by colonic IEL Vδ1 T, Vδ2 T, and ILC3 populations. (**F**) Pie charts depicting the relative expression of AHR, RORγt, and T-bet by Vδ1 T, Vδ2 T, and ILC3 populations in colonic IEL fraction from pre-SIV baseline through SIV infection and ART. Pie arcs represent expression of individual transcription factors, and pie slices represent the number of coexpressed transcription factors. Note that IEL data represent *n* = 5–6 animals because of insufficient cell yields from cryopreserved colon biopsies for these specific assays. The rare frequencies of IEL Vδ2 T and ILC3 populations precluded a meaningful analysis of their combinatorial transcription factor profiles at the post-ART time point. Data are shown as mean ± SEM and paired ANOVA test *P* values.

To further understand the gut-specific impacts of SIV infection and the influence of viral suppression with ART on γδ T cells and ILC3s, using the gating strategy shown in Figure 2A, we next examined their frequencies and immunophenotype in colonic lamina propria lymphocyte (LPL) and intraepithelial lymphocyte (IEL) compartments. Direct comparison between peripheral and mucosal compartments was restricted to the day 0, day 30, and week 12 (ART) time points to maintain a standardized sampling interval across all subjects and prioritize animal safety during the acute phase. As expected, a significant loss of CD4^+^ T cells was observed in colonic LPLs (*P* < 0.0001) and IELs (*P* < 0.01) by 6 weeks after SIV infection, resulting in near-complete depletion of CD4^+^ T cells in this gut compartment around the time of set-point viremia (Figure 4, A and D). Three months of daily ART partially restored CD4^+^ T cells in both IEL and LPL compartments (*P* < 0.05; Figure 4, A and D). However, CD4^+^ T cell frequencies in the LPLs remained significantly below baseline levels (*P* = 0.0058), indicating that viral suppression by ART promotes differential recovery of CD4^+^ T cells in the colonic lamina propria versus the epithelium.

In LPLs, Vδ2 T cells increased during acute infection and returned to baseline by 3 months after ART (*P* = 0.017), while Vδ1 T cells remained stable (Figure 4A). However, ILC3s mirrored CD4^+^ T cell kinetics, sharply declining at 1 month after infection (*P* < 0.0001) with partial ART-mediated restoration (Figure 4A). Notably, a transient Th17-type skewing, reflected by elevated RORγt, occurred in Vδ2 T cells and ILC3s during acute infection (*P* ≤ 0.012), whereas AHR remained unchanged across all 3 cell populations during acute SIV infection and after ART (Figure 4B). Progressive T-bet upregulation was noted in γδ T cells, most pronounced in Vδ1 (*P* = 0.0004), while T-bet^+^ ILC3s increased transiently during acute infection before returning to baseline (Figure 4B). These findings suggest that during acute SIV infection, Vδ2 T cells and ILC3s undergo transient Th17-type skewing in response to viral replication and CD4^+^ Th17 cell depletion in the gut (Figure 4C). Furthermore, the sustained T-bet upregulation in Vδ1 T cells, despite effective viral suppression with ART, points to a potential role for this subset in maintaining mucosal inflammation during chronic infection (Figure 4C).

The colonic IEL compartment exhibited a near-complete depletion of CD4^+^ T cells following SIV infection, accompanied by a reciprocal increase in CD8^+^ T cell frequencies (Figure 4D), highlighting a major shift in T cell composition at the mucosal barrier. Vδ2 T cells remained nearly undetectable at all time points, consistent with prior findings that Vδ1 T cells predominate among γδ T cells in the gut epithelium (21, 22). Similarly, lineage-negative CD127^+^ ILC3-like cells, a relatively rare subset in the IELs (23), showed a progressive and sustained decline after infection that was not reversed by ART (Figure 4D), suggesting poor restoration of this critical innate population in the epithelial niche. Interestingly, IEL-resident Vδ1 T cells increased significantly by 3 months after SIV+ART (*P* = 0.046), pointing to a selective expansion or enhanced retention of this subset in the epithelial compartment (Figure 4D). Functionally, RORγt-expressing Vδ1 T cells were significantly elevated during acute infection (*P* = 0.0025), mirroring the Th17-like skewing observed in Vδ2 T cells in the LPLs (Figure 4, E and F). However, this increase in RORγt expression was transient and declined to below baseline levels by 3 months after ART (Figure 4E). Because of the limited cellular yield from longitudinal colon biopsies, transcription factor analysis was prioritized for the diet-supplemented cohort (*n* = 5; Figure 4, B and E) to characterize the functional profile following intervention. The low abundance of ILC3s precluded a meaningful analysis of their transcription factor profiles in the IELs. Thus, compartmentalized γδ T cell and TCR-negative innate cell responses were observed in the colonic lamina propria and epithelium during SIV infection.

### Th1/Th17 cytokine dysregulation and transcription factor correlates in colonic γδ T cells during ART-treated SIV infection.

**Figure 5:**
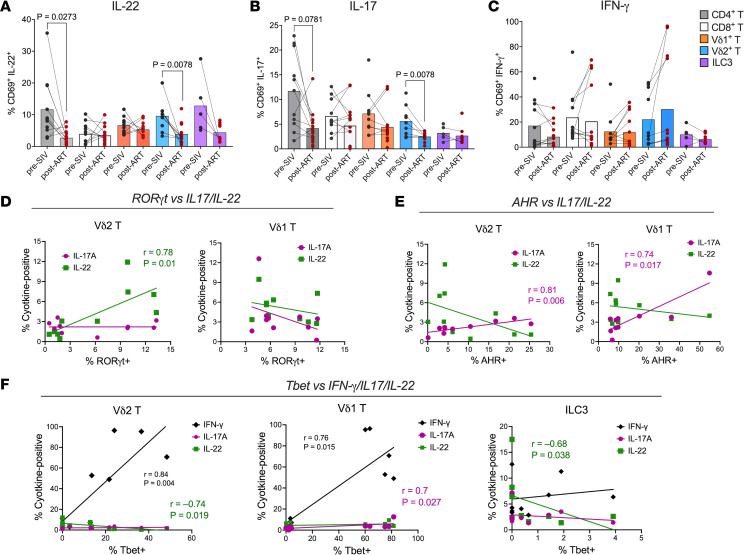
Cytokine production by colonic mucosal T cells and ILC3s during ART-suppressed SIV infection and association with lineage-defining transcription factors. Freshly isolated colonic LPLs were evaluated for cytokine production following ex vivo stimulation with PMA/calcium-ionomycin. (**A**–**C**) Intracellular expression of IL-22 (**A**), IL-17A (**B**), and IFN-γ (**C**) across γδ T cells, CD4^+^ T cells, CD8^+^ T cells, and ILC3s. Lines connect longitudinal samples from the same animal to illustrate shifts from pre-SIV baseline to ART-suppressed SIV infection. (**D**–**F**) Transcription factor correlations: correlation analysis between master regulators and cytokine output in colonic T cells and ILC3s. Transcription factor expression was measured in parallel and correlated with cytokine production. Correlations between RORγt (**D**) and AHR (**E**) expression and IL-17A or IL-22 production by colonic γδ T cell subsets are shown. Correlations between T-bet (**F**) and IFN-γ, IL-17A, or IL-22 production by colonic γδ T cells and ILC3s are presented. Spearman’s rank correlations were calculated using pooled samples from baseline and 30 days after infection to assess maintenance of these relationships during SIV-associated immune activation. For **A**–**C**, data represent mean ± SEM, with *P* values determined by Wilcoxon’s paired signed-rank test. For **D**–**F**, Spearman’s rank correlations were calculated using pooled longitudinal data to define the relationship between transcription factors and cytokines across healthy and SIV-associated immune activation.

To assess how RORγt, AHR, and T-bet expression affects gut γδ T cell and ILC3 function, we measured cytokine production in colonic LPLs after ex vivo mitogen stimulation. Consistent with our earlier findings (9), ex vivo production of Th17-type cytokines, IL-17A and IL-22, was markedly impaired during SIV infection despite ART, whereas IFN-γ production in mitogen-stimulated cells remained largely intact, except for a significant decline in CD4^+^ T cells (Supplemental Figure 3D). In colonic LPLs, IL-22 was significantly reduced in CD4^+^ (*P* = 0.0002) and Vδ2 T cells (*P* = 0.002), with a trend in ILC3s (*P* = 0.059), and IL-17 was decreased in CD4^+^ (*P* = 0.005) and Vδ2 T cells (*P* = 0.007) (Figure 5, A and B).

Notably, IL-22 expression in ex vivo–stimulated Vδ2 T cells correlated positively with RORγt expression (*P* = 0.01; Figure 5D), whereas IL-17 production was associated with AHR expression in both Vδ1 (*P* = 0.017) and Vδ2 T cells (*P* = 0.007) (Figure 5E), highlighting distinct transcriptional dependencies for these cytokines. As expected, T-bet expression strongly correlated with IFN-γ production in colonic γδ T cells (*P* = 0.004 in Vδ2; *P* = 0.015 in Vδ1; Figure 5F). Strikingly, T-bet also negatively correlated with IL-22 in Vδ2 T cells and ILC3s (*P* = 0.019 and 0.038, respectively), consistent with the skewing toward an IFN-γ–dominant phenotype observed with increased T-bet and reduced RORγt in colonic LPLs (Figure 5C). Interestingly, IL-17 production in Vδ1 T cells correlated positively with T-bet, suggesting that IFN-γ/IL-17–coproducing Vδ1 T cells are regulated via integrated T-bet and AHR signaling. No significant correlation was observed between AHR or RORγt expression and cytokine production in ILC3s (Supplemental Figure 3). In summary, chronic SIV+ART infection reprograms mucosal γδ T cells and ILC3s toward a T-bet–driven, IFN-γ–biased phenotype with diminished IL-17 and IL-22 production.

### In vitro effects of AHR ligands on mitigating HIV-induced damage to colonic epithelial monolayers.

**Figure 6:**
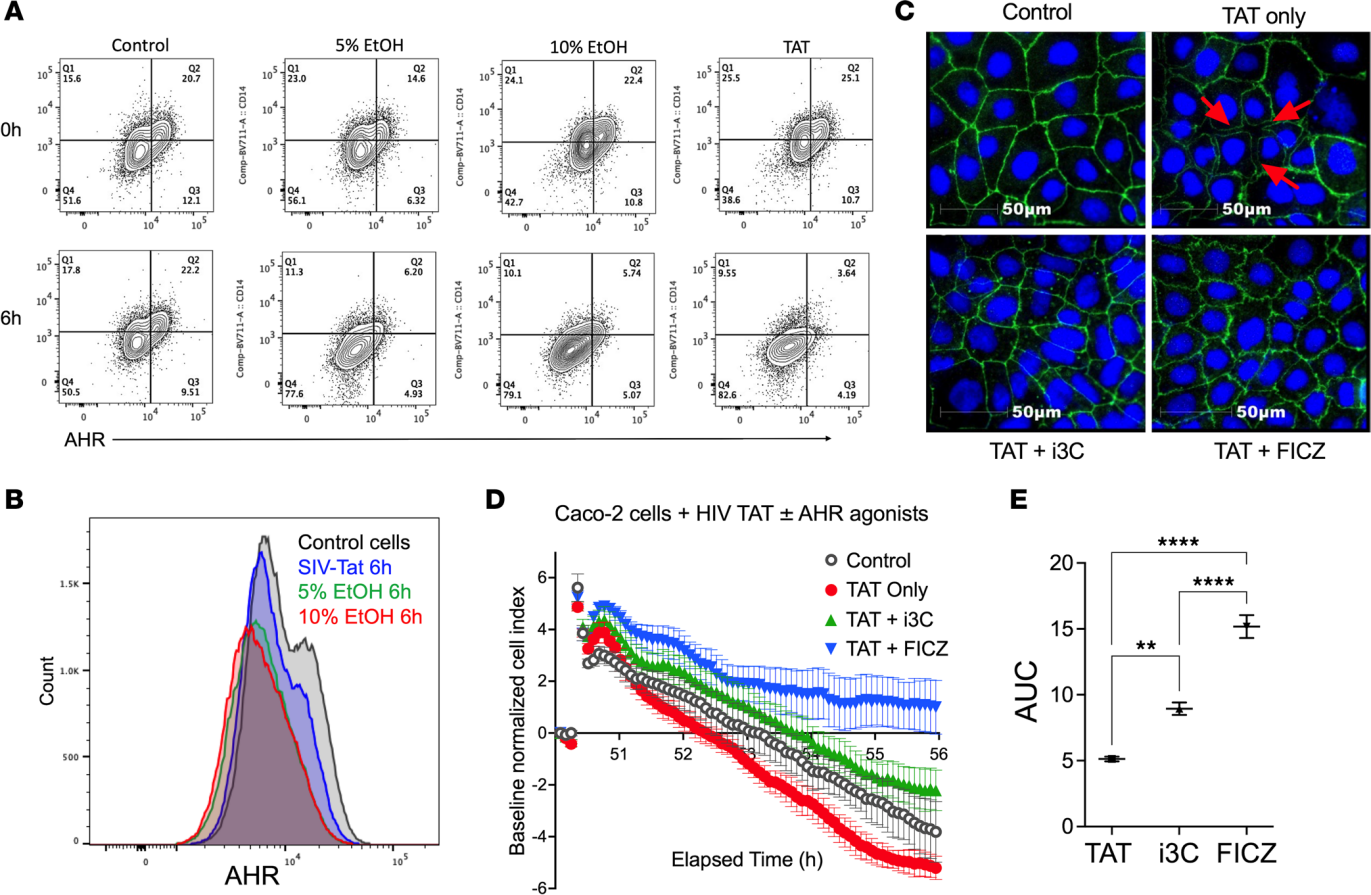
In vitro epithelial monolayer disruption and AHR downregulation by SIV Tat is reversed by ligand-specific activation through indoles. (**A**) Representative flow cytometry contour plots showing AHR expression in Caco-2 cells at 0 hours and 6 hours following exposure to control medium or medium supplemented with 5% EtOH, 10% EtOH, or 3 μM SIV Tat. (**B**) Representative histogram showing AHR expression in Caco-2 cells after 6 hours of exposure to EtOH- or Tat-containing medium compared with control conditions. (**C**) Representative immunofluorescence images of ZO-1 (green) and nuclei (DAPI, blue) in Caco-2 cells under control conditions (top left) or following 6 hours of treatment with Tat alone (top right), Tat plus i3C (bottom left), or Tat plus FICZ (bottom right). Scale bars: 50 μm. (**D**) Real-time cell analysis (RTCA) of Caco-2 monolayer integrity. Cells were seeded and allowed to form a confluent monolayer until the normalized cell index reached a stable plateau (50 hours after seeding), at which point HIV Tat alone or in combination with AHR agonists (i3C or FICZ) was added. Cell index was continuously monitored over the subsequent 6 hours and normalized to baseline at the time of treatment. Data are shown as mean ± SD of triplicate wells. (**E**) Area under the curve (AUC) analysis is shown as a single, quantitative value that denotes the cumulative cellular response over 6 hours, represented as mean ± SEM (**P < 0.005, ****P < 0.0001).

HIV proteins, including Tat, can disrupt intestinal epithelial integrity independent of viral replication (24). We treated differentiated human colonic epithelial Caco-2 monolayers with recombinant SIV Tat protein as an in vitro model. Tat exposure resulted in significantly reduced cell viability comparably to 5%–10% ethanol (Supplemental Figure 4), used as a well-established inducer of intestinal epithelial permeability. Further, SIV Tat decreased AHR expression (Figure 6, A and B), suggesting that Tat-mediated damage may occur, at least in part, via AHR suppression. Immunofluorescence staining for the tight junction protein ZO-1 revealed disrupted junctional morphology in Tat-treated Caco-2 monolayers, characterized by diminished ZO-1 signal intensity and visible intercellular gaps (Figure 6C, red arrows), indicative of compromised barrier integrity. Cotreatment with the AHR ligand 6-formylindolo[3,2-b]carbazole (FICZ) or indole-3-carbinol (I3C) preserved ZO-1 organization and restored the characteristic zigzag junctional pattern associated with well-polarized, mature epithelial monolayers (Figure 6C, bottom), with FICZ showing a stronger effect. Real-time cell analysis confirmed that Tat impaired barrier function (decreased impedance/cell index), which was significantly restored by FICZ and I3C, particularly FICZ (*P* < 0.0001), as evidenced by both real-time recovery kinetics and area under the curve analysis (Figure 6, D and E). Because cruciferous vegetables such as broccoli contain indole glucosinolates hydrolyzed into AHR agonists like indolocarbazole (ICZ) (25, 26), our subsequent experiments aimed to explore the in vivo effects of dietary indoles in modulating gut AHR signaling and intestinal epithelial barrier functions during ART-suppressed chronic SIV infection.

### Broccoli-based AHR ligand supplementation enhances epithelial barrier integrity, Vδ2 T cell function, and mucosal immune subset distribution during chronic SIV infection and ART.

**Figure 7:**
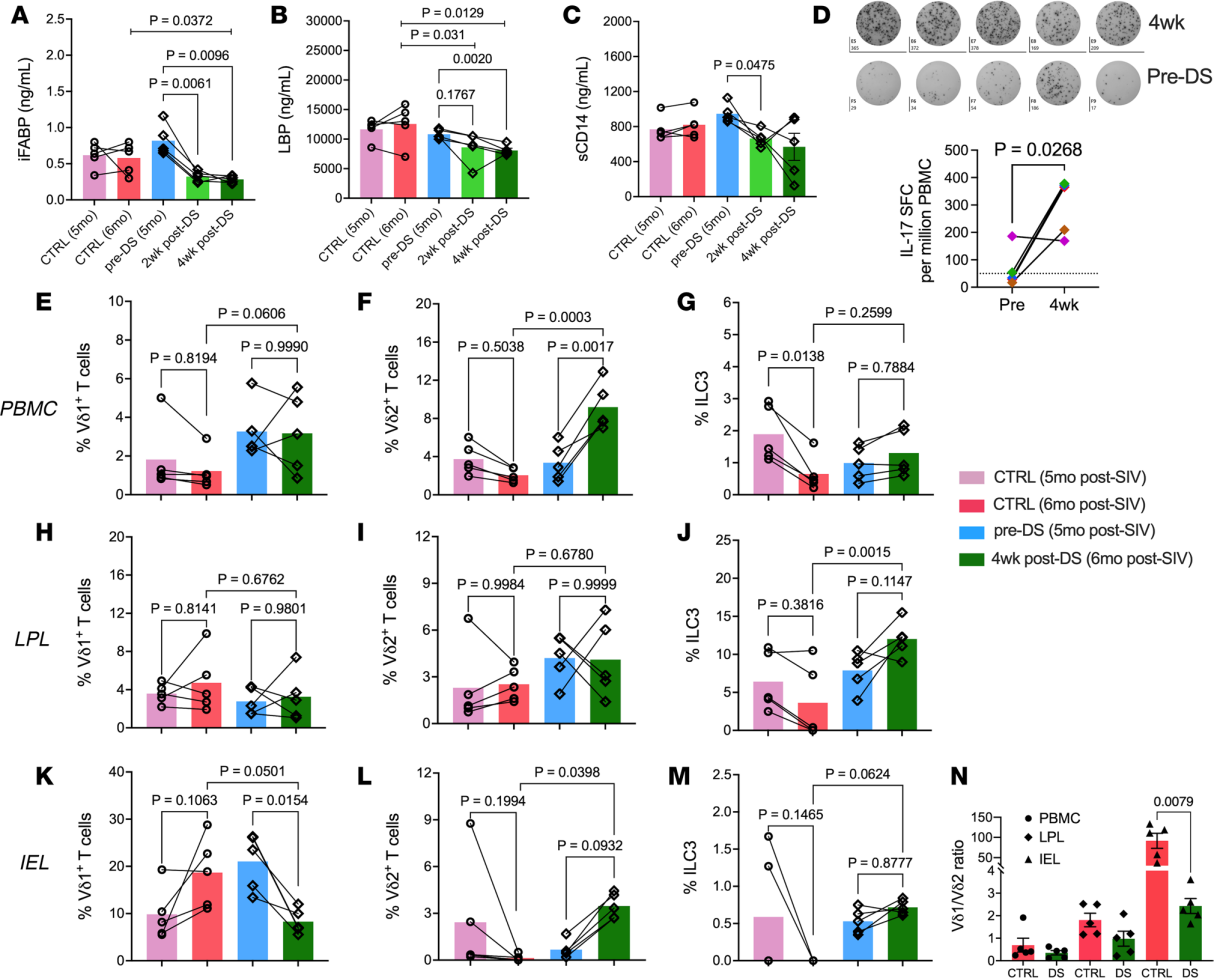
Effects of dietary indoles on plasma biomarkers and type 3 immune cell frequencies in chronically SIV-infected macaques on long-term ART. (**A**–**C**) Plasma iFABP (**A**), LBP (**B**), and sCD14 (**C**) at baseline and 2 and 4 weeks after supplementation and at baseline and 4-week-equivalent time points in control group. (**D**) IL-17^+^ PBMCs after Vδ2 T agonist stimulation at baseline and 4 weeks. (**E**–**M**) Frequencies of Vδ1 T cells, Vδ2 T cells, and ILC3s in PBMCs (**E**–**G**), LPLs (**H**–**J**), and IELs (**K**–**M**) from control versus DS groups. (**N**) Vδ1/Vδ2 ratios in PBMCs, LPLs, and IELs. For panel **N**, a Mann-Whitney test was used.

We next assessed the effects of short-term broccoli-based dietary supplementation (DS) as a physiologically relevant means of augmenting AHR ligand availability in the gut, on AHR signaling and intestinal inflammation in chronically SIV-infected macaques on long-term ART. At 5 months after SIV+ART, animals received approximately 30 μmol sulforaphane/Avmacol (2 tablets/day) for 2 weeks, escalating to approximately 60 μmol sulforaphane (4 tablets/day) for 2 more weeks. Plasma biomarkers showed reduced iFABP by 2 weeks and LPS-binding protein (LBP) by 4 weeks (Figure 7, A and B), indicating improved barrier integrity and decreased microbial translocation. Plasma sCD14 concentrations declined in three of the five animals by the two-week time point (Figure 7C). Concomitantly, DS enhanced IL-17 responses in circulating Vδ2 T cells, as demonstrated by an increase in IL-17 spot-forming cells following HMBPP stimulation of PBMCs (Figure 7D). Furthermore, at 4 weeks after DS, there was a coordinated trend toward reduced systemic inflammation, including TNF-α, the chemokines MCP-1 and CXCL13, and eotaxin, suggesting a broad systemic antiinflammatory effect. IL-1RA also declined, reflecting dampened negative feedback, while SDF-1α, FGF-2, and VEGF-D levels decreased, indicating reduced inflammatory cell recruitment and pro-angiogenic signaling (Supplemental Figure 5).

**Figure 8:**
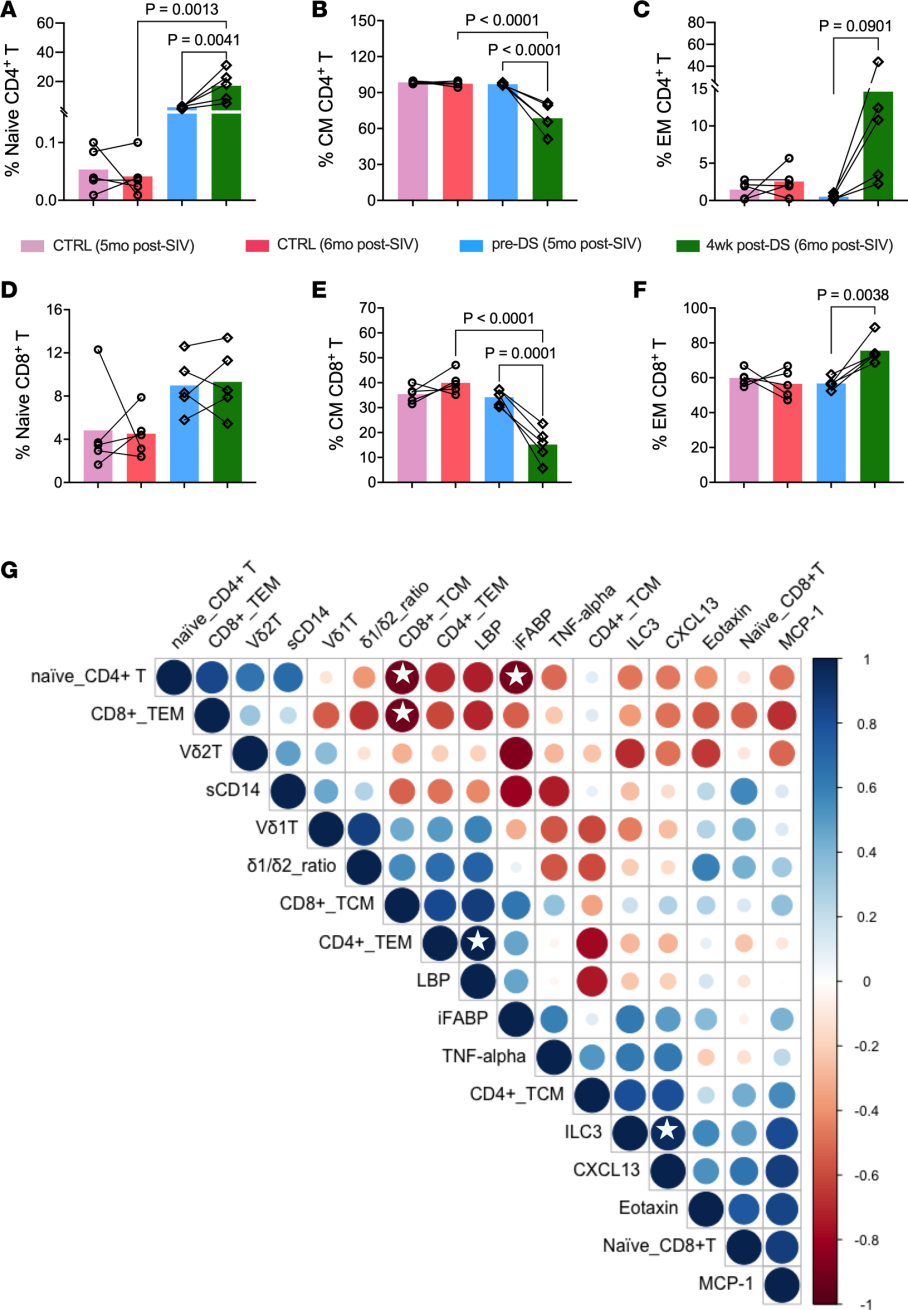
Impact of dietary indoles on colonic mucosal T cell memory distribution and its relationship to circulating inflammatory cytokines and barrier integrity. (**A**–**F**) Frequencies of CD95^–^CD28^+^ naive, CD95^+^CD28^+^ central memory (CM), and CD95^+^CD28^–^ effector memory (EM) subsets in colonic lamina propria CD4^+^ T cells (**A**–**C**) and CD8^+^ T cells (**D**–**F**). Data are shown as mean ± SEM. Longitudinal comparisons within the DS group across 3 time points were analyzed using 1-way repeated-measures ANOVA with Bonferroni’s post hoc correction. Comparisons between control and DS groups over time were performed using 2-way repeated-measures ANOVA to evaluate the interaction between time and intervention. (**G**) Pearson’s correlations from –1 (red) to +1 (blue); white stars indicate significance.

Flow cytometry showed a significant increase in circulating Vδ2 T cells at 4 weeks in the DS group, contrasting with significantly lower frequencies at the matching time point of 6 months after SIV in the control group (Figure 7F). Additionally, circulating ILC3 frequencies were maintained in the DS group, while there was a significant decline in the control group (Figure 7G). In colonic LPLs, although there were no significant changes in Vδ2 T cell frequencies both within and between groups, the DS group displayed higher ILC3 frequencies than the control group (Figure 7J). Notably, colonic IELs exhibited significantly reduced Vδ1 and increased Vδ2 frequencies (Figure 7, K and L) along with a trend of increased ILC3 frequencies (Figure 7M) in the DS group. Overall, these changes resulted in a lower Vδ1/Vδ2 ratio across all compartments, particularly in IELs (Figure 7N). Circulating Vδ1 T cell subsets (Figure 7E) and colonic LPL Vδ1 and Vδ2 T cell subsets (Figure 7, H and I) showed no significant differences in frequency. Classical T cell frequencies were unchanged in the colon (Supplemental Figure 6), but the DS group showed increased naïve CD4^+^ T cell frequencies (Figure 8A) and no change in CD8^+^ naïve cells (Figure 8D). Additionally, compared to the unchanged profiles of the control group, DS animals displayed significantly expanded effector memory (Figure 8C, F) and contracted central memory (Figure 8B, E) populations across both CD4^+^ and CD8^+^ T cell subsets.

Correlation analysis linked immune subsets with plasma biomarkers (Figure 8G). Naive CD4^+^ T cells negatively correlated with iFABP, consistent with their recovery after DS, and ILC3s tracked with CXCL13, suggesting a gut mucosal–B cell chemokine axis. MCP-1, CXCL13, and eotaxin clustered together, highlighting a shared inflammatory program spanning monocyte/macrophage activation, eosinophil recruitment, and lymphoid remodeling, which was dampened by DS (Supplemental Figure 5). Overall, DS improved epithelial barrier markers, enhanced IL-17–producing Vδ2 responses, and reshaped mucosal T cell and ILC3 subsets during chronic SIV+ART.

**Figure 9:**
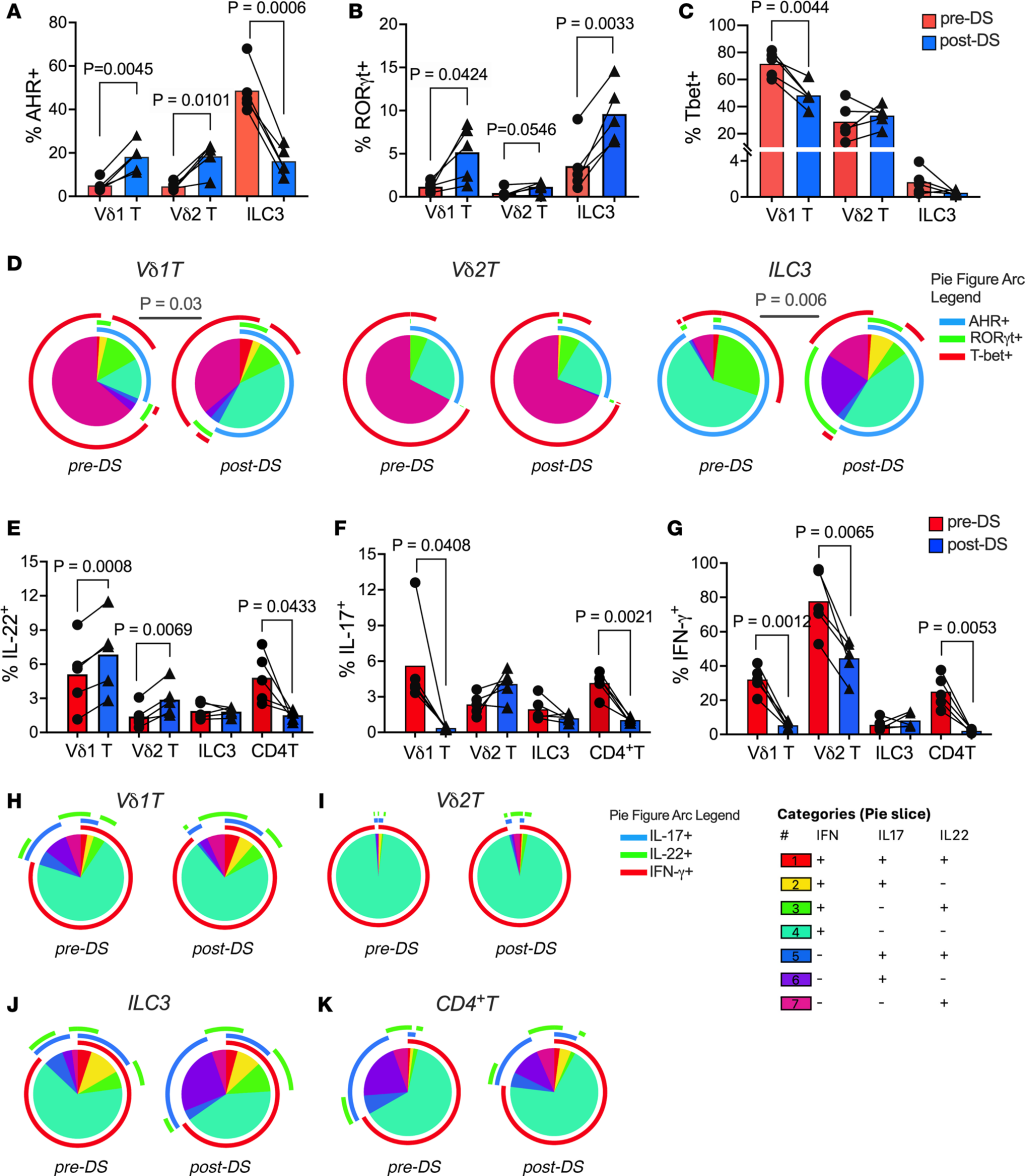
Effects of dietary indoles on transcription factor expression and cytokine-producing functions of colonic mucosal γδ T cells and ILC3s during chronic SIV+ART. Colonic LPLs were analyzed for transcription factor expression and cytokine production following PMA/ionomycin stimulation. (**A**–**C**) Frequencies of AHR (**A**), RORγt (**B**), and T-bet (**C**) expression were compared before and 1 month after dietary supplementation (DS) with indoles. (**D**) Pie charts depicting changes in the relative expression of AHR, RORγt, and T-bet by Vδ1 T, Vδ2 T, and ILC3 populations in colonic LPL fraction from pre- and post-DS. Pie arcs represent expression of individual transcription factors, and pie slices represent the number of coexpressed transcription factors. (**E**–**K**) Cytokine production of IL-22 (**E**), IL-17A (**F**), and IFN-γ (**G**) by Vδ1 T, Vδ2 T, ILC3, and CD4^+^ T cells was measured following mitogen stimulation of colonic LPLs, and combinatorial cytokine responses are shown in pie charts from before and after DS (**H**–**K**). Data are shown as mean ± SEM; paired *t* test values for comparisons between pre- and post-DS are shown.

In colonic LPLs, DS significantly increased AHR in Vδ2 T cells (*P* = 0.01) with a trend in Vδ1 T cells (*P* = 0.06) but decreased AHR in ILC3s (*P* = 0.006) (Figure 9A). RORγt increased across all subsets, most prominently in ILC3s (*P* = 0.003) and Vδ1 T cells (*P* = 0.04), while T-bet declined in Vδ1 T cells (*P* = 0.004) (Figure 9, B and C). These shifts indicate a move toward a type 3 immune phenotype, supported by polyfunctional transcription factor expression profiles in Vδ1 T cells and ILC3s (Figure 9D). Ex vivo stimulation showed increased IL-22 response in Vδ1 (*P* = 0.0008) and Vδ2 (*P* = 0.007), reduced IFN-γ, and unchanged IL-17A (Figure 9, E–G), with no significant changes in polyfunctional cytokine profiles (Figure 9, H–K). ILC3 cytokines were unaffected despite significantly increased RORγt expression. CD4^+^ and CD8^+^ T cells exhibited no functional recovery following supplementation. Their IL-17 and IL-22 responses continued to decline under chronic SIV+ART, consistent with progressive loss of epithelial barrier–protective functions, and were accompanied by a marked reduction in IFN-γ production (Figure 9G and Supplemental Figure 7), indicative of dampened inflammatory capacity. Taken together, these findings demonstrate that DS reprograms colonic γδ T cells toward type 3 immunity characterized by increased IL-22 production, a shift likely to enhance mucosal barrier integrity and epithelial repair.

**Figure 10:**
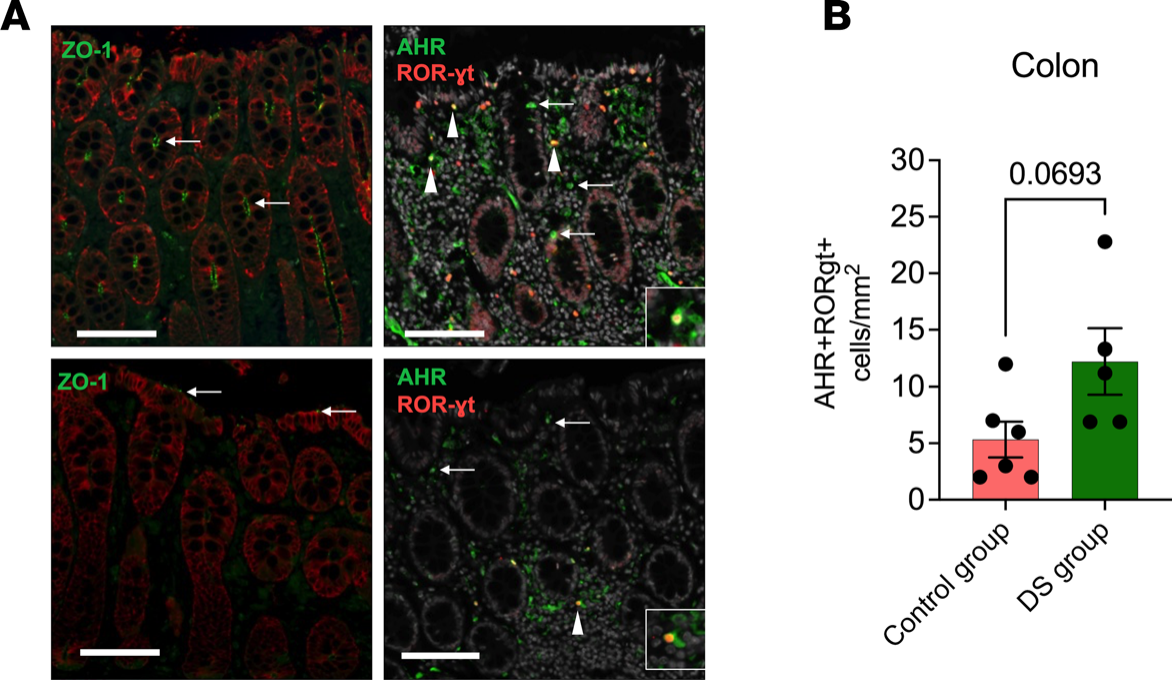
Enrichment of AHR/RORγt-coexpressing cells in dietary indole–supplemented animals during chronic SIV+ART. Fluorescent IHC demonstrating ZO-1, AHR, and RORγt expression in colon. (**A**) Representative colon sections from one SIV-naive (top) and one chronic SIV–positive macaque (bottom). The SIV-naive animal shows strong ZO-1 expression (green, arrows) along the apical margin of epithelial cells (red; top left), and abundant AHR-expressing (green, arrow) cells both with and without RORγt expression (red, arrowheads; top right). Inset highlights an AHR^+^RORγt^+^ cell in yellow. The chronically SIV-infected macaque demonstrates a loss of ZO-1 expression with only patchy, residual expression (green, arrows; bottom left), and a reduction in AHR-expressing cells within the mucosa and a depletion of single- and dual-positive RORγt-expressing cells (red, arrowhead; bottom right). Inset highlights an AHR^+^RORγt^+^ cell. Scale bars: 100 μm. Original magnification, ×200. White, DAPI (right); red, pan-cytokeratin (left), RORγt (right); green, ZO-1 (left), AHR (right). (**B**) AHR^+^RORγt^+^ cells/mm^2^ quantified using HALO software. Data show mean ± SEM and comparison between control and DS group by Mann-Whitney test.

To evaluate the localized impact of dietary indole supplementation on mucosal immunity, we used multiplex confocal immunohistochemistry (IHC) to quantify the density of AHR^+^RORγt^+^ cells within the colonic lamina propria. Representative images demonstrate that while SIV-naive animals maintained strong, continuous ZO-1 expression and abundant AHR^+^ and RORγt^+^ populations, chronic SIV infection resulted in the characteristic loss of apical ZO-1 and a depletion of these critical immune subsets (Figure 10A). Quantitative analysis revealed more than 2-fold higher mean density of dual-positive AHR^+^RORγt^+^ cells in the DS group compared with the control group (12.22 vs. 5.33 cells/mm^2^, respectively). While this comparison (Figure 10B) did not reach the threshold for traditional statistical significance (Mann-Whitney *P* = 0.0693), likely owing to the inherent constraints of the nonhuman primate cohort size (*n* = 5–6 per group), the greater AHR^+^RORγt^+^ cell density suggests a biological trend toward the enrichment of these regenerative immune subsets with the intervention. These data suggest that a higher prevalence of the AHR-RORγt axis may be a critical factor in the intestinal barrier restitution observed in the DS-treated cohort.

## Discussion

Despite effective ART, intestinal barrier dysfunction persists in people with HIV, underscoring gaps in our understanding of the mechanisms that sustain mucosal disruption. Our findings suggest that SIV infection and ART differentially shape epithelial integrity and mucosal immune regulation, with persistent defects in type 3 immunity pointing to AHR-dependent pathways as potential contributors to barrier homeostasis. Acute SIV infection was characterized by loss of ILC3s and Vδ2 T cells, along with compartment-specific alterations in the colon that reflected a localized Th1/Th17 imbalance and impaired epithelial-immune crosstalk. While ART partially reduced circulating markers of epithelial barrier disruption, it did not reverse SIV-driven Th1 polarization or restore type 3 immunity in the gut. Notably, AHR ligands attenuated SIV-induced epithelial injury in vitro, and dietary supplementation was associated with reduced barrier disruption and enhanced gut IL-22 responses, suggesting a mechanistic link between AHR signaling and the preservation of mucosal integrity during treated infection.

Our findings highlight that disruption of type 3 immunity is a central barrier to mucosal repair in HIV/SIV infection. While loss of CD4^+^ T cells has long been recognized as a hallmark of disease, consistent with prior reports, our data point to a broader defect involving γδ T cells and ILC3s, populations critical for sustaining epithelial barrier integrity. The transient expansion and gut trafficking of Vδ2 T cells during acute infection suggest an early compensatory response, but one that is neither durable nor sufficient under ART. This dynamic may explain why epithelial tight junction restoration remains incomplete, as reflected by persistent elevations in zonulin despite reduced iFABP levels (27–29). These results align with growing evidence that innate lymphocyte dysfunction underlies the failure of ART to fully normalize gut barrier function and inflammation (7, 14, 30). Importantly, they suggest that interventions aimed at preserving or restoring type 3 immunity, whether by supporting γδ T cell and ILC3 function or by modulating barrier regulatory pathways, may be essential to achieve durable mucosal immune homeostasis. More broadly, our study reinforces the concept that immune restoration in HIV/SIV is not simply a matter of CD4^+^ T cell recovery but requires a coordinated reconstitution of multiple mucosal immune cell networks that protect and repair the epithelial barrier.

In SIV-naive macaques, colonic immunity is dominated by AHR^+^RORγt^+^ γδ T cells and ILC3s, consistent with their roles in mucosal defense and tissue repair. However, following SIV infection, impaired IL-17A and IL-22 production by these cells correlated with reduced RORγt and AHR expression, revealing a mechanism by which SIV, despite ART, compromises gut barrier function. Our data further reveal that chronic SIV+ART reprograms γδ T cells and ILC3s toward a T-bet–driven, IFN-γ–biased phenotype, at the expense of IL-17 and IL-22. Although elevated T-bet supports HIV-specific CD8^+^ T cell cytotoxicity (31) and may promote IL-22–producing ILCs (32), production of IL-22 and IL-17 by intestinal immune cells is essential for epithelial integrity, antimicrobial defense, and microbiome homeostasis (33, 34), and their loss likely sustains barrier dysfunction and microbial translocation during chronic HIV/SIV infections. The reciprocal regulation of IL-22 by RORγt and T-bet, and of IL-17 by AHR and T-bet, underscores the transcriptional plasticity of γδ T cells and ILC3s in mucosal inflammation (35, 36). Notably, intraepithelial ILC3s remain persistently depleted despite ART, indicating durable disruption of epithelial immune integrity. Together, these findings suggest that transcriptional reprogramming of mucosal type 3 effector lymphocytes is a critical barrier to gut barrier restoration and identify RORγt and AHR signaling as potential therapeutic targets to reestablish mucosal immunity in treated HIV infection.

HIV-associated dysbiosis perturbs tryptophan metabolism and reduces microbiota-derived indoles, linking microbial imbalance to impaired type 3 immunity (6, 37). Interventions aimed at restoring mucosal function have demonstrated that probiotics improved CD4^+^ T cell recovery and reduced inflammation in SIV-infected macaques, particularly with IL-21 supplementation (38, 39), while fecal microbiota transplantation lowered gut permeability in ART-suppressed people with HIV (40). Diet quality also influences mucosal immunity, with Mediterranean diet improving immune activation and microbiota composition (41), whereas high-fat diet accelerated disease progression and microbial translocation (42).

Because chronic HIV/SIV alters the microbiome and may impair microbial conversion of glucobrassicin into the AHR ligand I3C, we used a broccoli-based supplement with active myrosinase to release I3C independent of microbial metabolism. Critically, significant reductions in the IEBD biomarkers iFABP and LBP were observed exclusively within the diet supplement group. In sharp contrast to controls, this intervention ameliorated mucosal barrier dysfunction during treated SIV infection by reconstituting γδ T cell and ILC3 populations. These structural improvements were further supported by an expansion of colonic naive CD4^+^ and effector memory CD8^+^ T cells, alongside a significant contraction of Vδ1/Vδ2 ratios in both systemic and intestinal compartments. The anatomical distribution of Vδ1 and Vδ2 T cell subsets is highly compartmentalized in nonhuman primates, with higher Vδ1/Vδ2 ratios in the gut and spleen than in peripheral blood or lymph nodes, a balance that is significantly altered during SIV infection toward increase in Vδ1 T cells (43). Vδ1/Vδ2 inversion in both peripheral blood (44, 45) and the gastrointestinal tract (46) is a hallmark of HIV infection, which is driven in part by loss of gut mucosal Vδ2 cells and expansion of Vδ1 T cells. This inversion correlates with elevated intestinal barrier dysfunction biomarkers and systemic inflammation during long-term ART (9). Importantly, similar Vδ1/Vδ2 imbalances are observed in other chronic inflammatory conditions affecting the gut, such as kidney disease, viral hepatitis, and obesity (8), highlighting a broader link between γδ T cell subset dysregulation, gut permeability, and chronic inflammation. Thus, the observed rebalancing in the DS group in our study suggests a mechanism for barrier protection, consistent with murine studies in which I3C activated AHR, induced its target gene, Cyp1a1, restored intestinal γδ T cells (47), and promoted IL-22–mediated epithelial repair (18), effects abrogated by IL-22 blockade (48). Notably, IL-22 upregulation occurred with I3C and not with butyrate, indicating mechanisms beyond butyrate production, since butyrate alone did not induce IL-22 expression. Consistently, butyrate supplementation in SIV-infected macaques failed to reduce microbial translocation or inflammation (49), likely because of its predominant GPR-dependent rather than AHR-mediated signaling. These results establish a translational bridge between murine and primate gut immunity, highlighting the central role of the gut AHR pathway in SIV-induced barrier disruption.

Our findings indicated that dietary indole supplementation was associated with a trend of reduction across several classes of circulating inflammatory mediators, specifically lowering key cytokines, chemokines, and angiogenic factors like TNF-α, MCP-1, CXCL13, and VEGF-D alongside significant lowering of IEBD biomarkers exclusively within the DS group. This suggests that the supplements dampened immune activation and tissue remodeling pathways. Correlation analyses further revealed that the supplements attenuated a chemokine-driven inflammatory axis (MCP-1, CXCL13, eotaxin) while promoting gut barrier repair and mucosal immune balance. We also observed a restoration of naive CD4^+^ T cells, and an association between ILC3s and CXCL13, pointing to integrated T cell and innate lymphoid contributions to gut immune remodeling. Though not statistically significant in this small cohort, trends toward reduced IL-1RA and SDF-1α suggest a decreased need for compensatory antiinflammatory responses. Importantly, we observed that macaques receiving dietary indole supplementation exhibited a more than 2-fold higher mean density of dual-positive AHR^+^RORγt^+^ cells in the colonic mucosa compared with the control group. While the cross-sectional nature of this comparison precludes a definitive claim of cellular expansion within individual animals, the marked shift in the immunological profile of the DS group suggests that indole-driven AHR activation favors the enrichment of these regenerative subsets during chronic SIV infection. Collectively, our data demonstrate that the dietary indoles act through a multipronged mechanism to preserve intestinal homeostasis during treated SIV infection. By specifically augmenting the ILC3–γδ T cell axis, the intervention provides the necessary cytokine signaling to maintain epithelial tight junctions, thereby preventing the translocation of microbial products as evidenced by the exclusive reduction of iFABP and LBP in the supplement group. Furthermore, the expansion of naive CD4^+^ and effector memory CD8^+^ T cells in the colon, alongside the normalization of the Vδ1/Vδ2 ratio, suggests a shift from a chronically exhausted immune profile toward an immunocompetent state capable of mitigating systemic inflammation.

The pleiotropic nature of the AHR necessitates a careful distinction between its various ligands. While high-affinity signaling induced by persistent organic pollutants, such as dioxins, is associated with toxicological and pro-carcinogenic off-target effects (50, 51), dietary indoles function as transient, low-affinity physiological agonists with a significantly lower risk of systemic toxicity (52). Unlike persistent xenobiotics, indole-derived ligands undergo rapid metabolic clearance, thereby preventing the sustained transcriptional hyperactivation that drives pathological outcomes. Notably, the primary objective of this study was to characterize the mechanistic capacity for gut-specific AHR modulation rather than to evaluate sulforaphane as a definitive clinical therapy for intestinal barrier disruption in people living with HIV. Supporting the translational safety of this approach, a recent 12-week clinical trial in virally suppressed people living with HIV demonstrated that sulforaphane supplementation at similar doses was well tolerated and significantly reduced C-reactive protein levels (53). Nevertheless, the long-term oncogenic and systemic safety of chronic indole supplementation in people living with HIV, a population with unique immune surveillance challenges, requires further longitudinal characterization. Future clinical investigations should prioritize pharmacokinetic monitoring and tissue-specific activation profiles to precisely define the therapeutic windows that maximize mucosal restoration while minimizing systemic risk. It is important to note that indole supplementation in this study was used primarily as a mechanistic tool to probe the importance of the AHR signaling pathway in the context of ART-suppressed chronic SIV infection. While this intervention addresses a metabolic deficiency resulting from microbial dysbiosis rather than reconstituting the microbial community itself, our findings suggest that dietary indole supplementation may compensate for the metabolic deficits associated with SIV-induced dysbiosis. These data indicate that the host AHR–RORγt–IL-22 axis remains functionally responsive and may be targeted to support mucosal repair, even in the presence of an altered microbial community.

While our study provides insights into the role of AHR-RORγt signaling in gut epithelial barrier repair during chronic ART-suppressed SIV infection, there are certain limitations. First, the small sample size (*n* = 5–6 per group) is a constraint inherent to nonhuman primate studies; however, the use of longitudinal sampling within the DS group allows for high-confidence internal controls that minimize the impact of inter-animal variability by tracking changes from baseline. Second, while the biomarker and flow cytometry data capture these temporal shifts, the IHC analysis of AHR^+^RORγt^+^ cells was performed cross-sectionally at the endpoint. Although this precluded the direct visualization of cellular kinetics within the colonic tissue over the course of treatment, the substantial differences in mean cell density between the groups provide critical spatial validation. These localized findings corroborate our longitudinal systemic data and gut cellular composition profiles, offering a high-resolution snapshot of the tissue-level environment following indole supplementation. Finally, further clinical trials are necessary to confirm whether these AHR-mediated mechanisms translate directly to improved clinical outcomes in people living with HIV on long-term ART.

In conclusion, our study provides proof of principle that dietary indoles can modulate gut mucosal immunity during SIV-induced barrier disruption. While I3C is likely the primary bioactive compound, other metabolites such as sulforaphane may contribute, and microbiome effects were not prospectively assessed. Nonetheless, these findings highlight a potential role for the ILC3/γδ T cell compartment and AHR/IL-22–associated pathways in intestinal barrier maintenance and repair during ART-suppressed SIV infection. The observed associations between type 3 immunity, the AHR-RORγt axis, and epithelial function generate testable hypotheses. Future studies, including spatial transcriptional profiling, will be important to define how microbiota- and diet-derived ligands influence mucosal repair and barrier homeostasis.

## Methods

### Sex as a biological variable.

Our control cohort is a part of previous studies that used only female macaques. However, we have observed similar temporal dynamics of the immune cell subpopulations and gut barrier disruption in male macaques in our other cohorts using identical SIV infection and ART regimen.

### Animals, viral inoculation, and ART.

Eleven healthy adult Indian-origin rhesus macaques (*Macaca mulatta*), aged 5–10 years and seronegative for SIV, HIV-2, STLV-1, SRV-1, and herpes B, were used in this study. To establish a robust and reproducible baseline for the evaluation of dietary supplementation (DS), we integrated longitudinal data on viral loads, plasma biomarkers, and circulating T cell frequencies during acute SIV infection and short-term ART from a previously characterized control cohort (*n* = 6) (9) with our current intervention group (*n* = 5). While basic T cell frequencies, plasma biomarkers, and viral loads for the *n* = 6 cohort were previously reported, all secondary immunophenotyping and functional assays, including ILC3 data, expression of AHR, RORγt, and T-bet in all immune subpopulations, cytokine production, and homing markers, were generated specifically for this study using cryopreserved samples to provide a comprehensive characterization of these subsets. All animals were subjected to identical experimental protocols: Animals were infected intrarectally with 2,500 TCID_50_ SIVmac251 (Preclinical Research and Development Branch, National Institute of Allergy and Infectious Diseases). ART was administered daily via subcutaneous injection: 5.1 mg/kg tenofovir disoproxil fumarate, 30 mg/kg emtricitabine, and 2.5 mg/kg dolutegravir in 15% kleptose solution at pH 4.2 (9). Plasma viral loads were quantified using the Roche High Pure Viral RNA Kit (54). Beginning 5 months after SIV+ART, animals in the DS group received a 4-week dose-escalation course of Avmacol (Nutramax Laboratories). The regimen consisted of 2 tablets daily for the first 2 weeks, followed by 4 tablets daily for the final 2 weeks. Each tablet contained 125 mg of broccoli seed powder and 50 mg of broccoli sprout extract, yielding approximately 15 μmol of sulforaphane. Total daily sulforaphane intake was approximately 30 μmol during the initial phase and approximately 60 μmol during the escalation phase. This time frame corresponds with the turnover rate of the intestinal epithelium and provides sufficient time to observe the recruitment and maturation of T cell and ILC3 subsets within the gut-associated lymphoid tissue. To ensure that observed biological effects were attributable specifically to the defined dose of broccoli extract and minimize background indole intake, all cruciferous vegetables, the primary sources of glucoraphanin-derived indoles, were strictly excluded from the enrichment diets of all animals for the duration of the study. Non-cruciferous items (e.g., carrots, apples) were retained to support animal health.

### Cell isolation.

Blood collected in EDTA tubes (Sarstedt) was processed immediately. PBMCs were isolated by density gradient centrifugation (Lymphocyte Separation Medium, MP Biomedicals) at 500*g* for 45 minutes for phenotyping and functional assays. Colonic intraepithelial lymphocytes and lamina propria lymphocytes (LPLs) were isolated as described (55). Briefly, biopsies were washed with PBS, epithelial cells removed with RPMI 1640 plus 5% FBS and 5 mM EDTA at 37°C for 1 hour, and tissue digested with RPMI-5 plus 60 U/mL type II collagenase. LPLs were enriched via Percoll density gradients, washed, and resuspended in RPMI-10 plus 10% FCS. Cell viability was greater than 90% by trypan blue exclusion.

### Immunophenotyping.

Polychromatic flow cytometry was performed using anti-human mAbs cross-reactive with rhesus macaques (9). Fresh or frozen PBMCs (1 million to 2 million) were stained with viability dye, surface markers (Supplemental Table 1), and the transcription factors RORγt, T-bet, and AHR after fixation (True Nuclear Kit, BioLegend). Unstained controls were included with each batch. Cells were resuspended in PBS plus 1% formaldehyde, and at least 200,000 lymphocytes were acquired on a BD Symphony (BD Biosciences); analysis was performed using FlowJo v10 (FlowJo).

### Plasma markers of inflammation, microbial translocation, and intestinal damage.

Frozen plasma was thawed, filtered (Ultrafree, Millipore), and used for multiplex quantification of 37 cytokines, chemokines, and growth factors (ProcartaPlex, Invitrogen) per the manufacturer’s instructions. Data were acquired on a Bio-Plex 200 and analyzed with Bio-Plex Manager v6.1. Plasma iFABP, LBP, sCD14, and zonulin were measured using commercial ELISA kits (MyBioSource, R&D Systems, Alpco Diagnostics) in duplicate, and data were analyzed with Gen5 software (BioTek).

### Functional analyses.

PBMCs and rectal LPLs were resuspended at 1 × 10^6^ cells/mL in RPMI plus 10% FBS and antibiotics, then stimulated 16 hours at 37°C with PMA/ionomycin with or without brefeldin A. Cells were stained for surface (CD45, CD3, CD4, CD8, TCR γδ/Vδ1/Vδ2, CD161) and intracellular markers (CD69, IL-17, IL-22, TNF-α, IFN-γ) (Supplemental Table 2), fixed, and acquired on a BD Symphony/LSRFortessa; analysis was via FlowJo v10. IL-17–secreting cells were measured by ELISPOT using PMA/ionomycin or HMBPP for γδ T cells; responses more than 2-fold above background and greater than 50 SFC/10^6^ PBMCs were considered positive.

### Effect of SIV Tat on Caco-2 epithelial barrier integrity monitored by real-time cell analysis.

Caco-2 cells (ATCC HTB-37) were seeded at 20,000 cells per well on flat-bottomed, 96-well tissue culture plates in DMEM with 20% FBS, 1% non-essential amino acids, and antibiotics. Caco-2 monolayers were treated with medium containing 5% or 10% (vol/vol) ethanol for up to 24 hours to serve as positive controls for monolayer disruption and the modulation of tight junction proteins (56). Cell adhesion was monitored in real time using impedance-based real-time cell analysis (RTCA; xCELLigence, Agilent Technologies), with cell index (57) normalized to pretreatment values. After about 48 hours, cells were treated with medium and 3 μM SIVmac_239_ Tat (ARP-12765, NIH HIV Reagent Program), with or without I3C (1 μg/mL) or FICZ (1 μg/mL) (Sigma-Aldrich product 17256, SML1489), and cell index was measured every 5 minutes in quadruplicate as previously described (58). SIV Tat was used in human Caco-2 cells owing to its conserved structure and function with HIV-1 Tat (59, 60), enabling correlation of in vitro effects with SIV-infected macaque mucosa. For ZO-1 immunofluorescence, parallel monolayers in glass-bottomed plates were treated 5 hours, washed, fixed with 2% PFA, permeabilized with 0.2% Triton X-100, and blocked with 5% goat serum. Cells were incubated with anti–ZO-1 primary antibody (1:100) overnight, followed by Alexa Fluor 488 secondary (1:500) and DAPI nuclear stain. Imaging was performed on a Nikon Eclipse Ti2 microscope, and ZO-1 junctional integrity was qualitatively assessed.

### Immunohistochemistry and immunofluorescence.

IHC was performed as previously described using the antibodies (Supplemental Table 3) targeting ZO-1 (1:1,000; clone ZO1-1A12), cytokeratin (1:400; clone AE1/AE3), AHR (1:1,000; rabbit polyclonal), RORγt (1:100; clone 6F3.1), and DAPI (1:20,000). Colonic tissues (4 μm FFPE sections) were mounted on Superfrost Plus slides, baked at 60°C, deparaffinized in xylene, and rehydrated through graded ethanol. Heat-induced epitope retrieval was performed via microwave in Tris-based buffer (pH 9.0; Vector Laboratories) with 0.01% Tween 20 for 20 minutes. Sections were subsequently equilibrated in citrate buffer (pH 6.0; Vector Laboratories). Nonspecific binding was mitigated using a tiered blocking strategy: protamine sulfate (16 mg/mL), Background Punisher (Biocare Medical), and serum-free protein block (Agilent Technologies). For the initial detection panel, mouse anti-RORγt primary antibody was incubated for 60 minutes, followed by signal amplification using the MACH3 AP polymer system (Biocare Medical). Chromogenic development was executed with ImmPACT Permanent Red (Vector Laboratories). Subsequent AHR detection and a secondary structural panel (ZO-1 and pan-cytokeratin) were performed using a Ventana Discovery Ultra autostainer. To ensure signal fidelity between targets, an intermediate heat denaturation step was employed. Automated staining parameters included 32-minute primary antibody incubations and 16-minute secondary antibody incubations at 37°C. Fluorochromes (rhodamine and Cy5) and DAPI counterstain were applied for 8 minutes each. After automated processing, slides were cleared of residual mounting oils using a dilute aqueous surfactant, rinsed in deionized water, and mounted in an anti-fading Mowiol/DABCO medium (in-house; see ref. 61 for recipe). Whole-slide digital imaging was performed on a Zeiss Axio Slide Scanner. Quantitative spatial analysis of AHR^+^RORγt^+^ cell density was conducted by a board-certified pathologist using HALO software (Indica Labs).

### Statistics.

Data were analyzed using GraphPad Prism (version 10.0). As appropriate, unpaired or paired 2-tailed *t* tests and 2-way ANOVAs or mixed-effects models were used in statistical analyses of plasma analyte concentrations, lymphocyte phenotype, and function frequencies. Longitudinal changes within the DS group across 3 time points (baseline, 2 weeks, 4 weeks) were evaluated using a 1-way repeated-measures ANOVA followed by Bonferroni’s post hoc test for pairwise comparisons. To determine whether observed changes were a direct result of the therapy rather than natural longitudinal fluctuations (time effects), comparisons between the control and DS groups were performed using a 2-way repeated-measures ANOVA. The interaction *P* value (time × intervention) was used to statistically confirm that the trajectory of immune parameters in the DS group was distinct from the control cohort. For non-parametric data, Wilcoxon’s signed-rank test was used. Correlation analyses were performed using Pearson’s correlation coefficient. In all cases, *P* < 0.05 was considered statistically significant. Significance of polyfunctional cytokine expression was assessed using the SPICE test on relative expression values. Correlations were calculated in R using the Hmisc package and visualized with corrplot. To validate the integration of the control cohort (*n* = 6) with the current intervention cohort (*n* = 5), we performed a sensitivity analysis comparing baseline physiological and virological parameters (animal characteristics summarized in Supplemental Table 4). No significant differences were observed between the 2 independent groups in preinfection CD4^+^ T cell frequencies (Mann-Whitney *U* test, *P* > 0.05) or peak plasma viral load magnitudes (day 14 post-SIV; *P* > 0.05).

### Study approval.

All animal studies were approved by the Tulane University Institutional Animal Care and Use Committee, and all animals were born and housed at the Tulane National Biomedical Research Center in accordance with Association for Assessment and Accreditation of Laboratory Animal Care International (AAALAC) standards. Animal housing and studies were carried out in strict accordance with the recommendations in the *Guide for the Care and Use of Laboratory Animals* of the National Institutes of Health (NIH; National Academies Press, 2011; AAALAC 000594) and with the recommendations of the Weatherall report: The Use of Non-Human Primates in Research.

### Data availability.

Values for all data points in graphs within the main article figures and the supplemental material are reported in the Supporting Data Values file.

## Author contributions

ST wrote the original draft of the manuscript, developed methodology, and performed formal analysis, data curation, and conceptualization. RVB, CCM, and ARVZ developed methodology and performed investigation and formal analysis. ABM and IB reviewed and edited the manuscript. VAH developed methodology and performed data curation. LADM performed investigation. DAW reviewed and edited the manuscript and performed conceptualization. AGM reviewed and edited the manuscript and performed investigation. NR reviewed and edited the manuscript, supervised the study, and performed project administration, funding acquisition, and conceptualization.

## Conflict of interest

The authors have declared that no conflict of interest exists.

## Funding support

This work is the result of NIH funding, in whole or in part, and is subject to the NIH Public Access Policy. Through acceptance of this federal funding, the NIH has been given a right to make the work publicly available in PubMed Central.

- NIH grants R01DK131930 (to NR), R56DK131531 (to NR), and P20GM103629 (to NR).
- NIH P51OD011104 (base grant for Tulane National Biomedical Research Center).

## Supplementary Material

Supplemental data

Supporting data values

## Version 1 04/02/2026

In-Press Preview

## Version 2 05/22/2026

Electronic publication
