# Supplementary material for: Dietary indoles influence the AHR-RORγt axis and mucosal immune homeostasis in ART-treated SIV infection
Source: JCI Insight. 2026 Apr 2;11(10):e201258. doi: 10.1172/jci.insight.201258 (PMC13232730; doi:10.1172/jci.insight.201258)
Supplement: Supplemental data [file jciinsight-11-201258-s196.pdf]

Supplemental Material

Title: Dietary Indoles Influence the AHR–RORγt Axis and Mucosal Immune Homeostasis in ART-Treated SIV Infection

Siva Thirugnanam[1], Alison R. Van Zandt[1], Alexandra B. McNally[1, 2], Victoria A. Hart[1], Isabelle Berthelot[1], Cecily C Midkiff[1], Lara A. Doyle-Meyers[1], David A. Welsh[3], Robert V Blair[1], Andrew G. MacLean[1, 2, 4, 5], Namita Rout[1, 2, 5*]

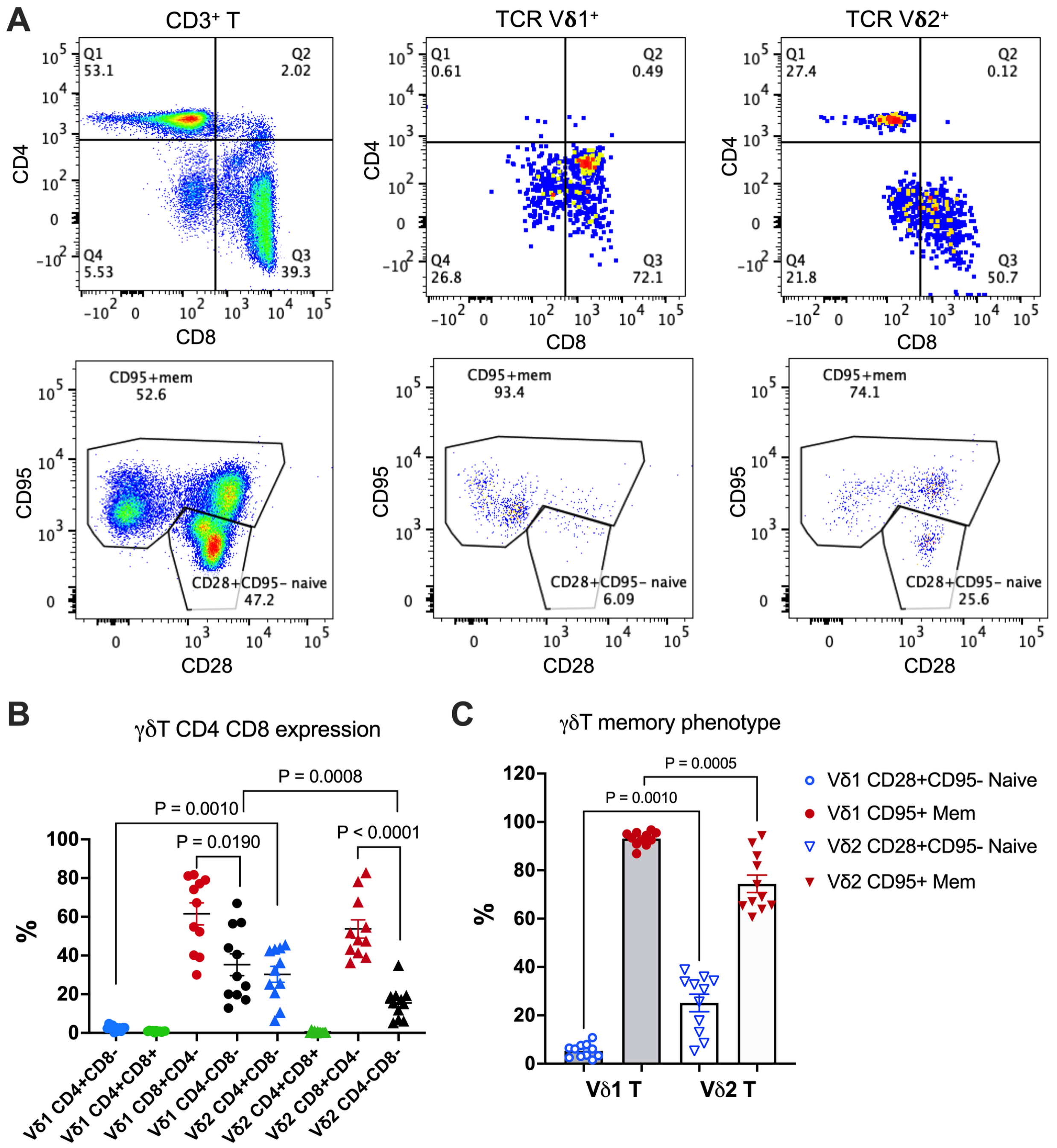

Supplemental Figure 1. CD4 and CD8 expression and memory phenotype of circulating Vδ1$^+$ and Vδ2$^+$ T cells. **(A)** Representative flow cytometry plots showing expression of CD4, CD8, CD28, and CD95 on T cell subpopulations. **(B)** Percentages of CD4$^+$CD8$^-$, CD4$^+$CD8$^+$, CD4$^+$CD8$^-$, and CD4$^-$CD8$^-$ cells in Vδ1$^+$ and Vδ2$^+$ T cells in PBMCs at pre-SIV baseline. **(C)** Percentages of CD28$^+$CD95$^-$ naïve cells and CD95$^+$CD28$^-$ memory cells in PBMC Vδ1$^+$ and Vδ2$^+$ T cells pre-SIV baseline. Data show Mean ± SEM and P values for each comparison Wilcoxon matched-pairs signed rank test.

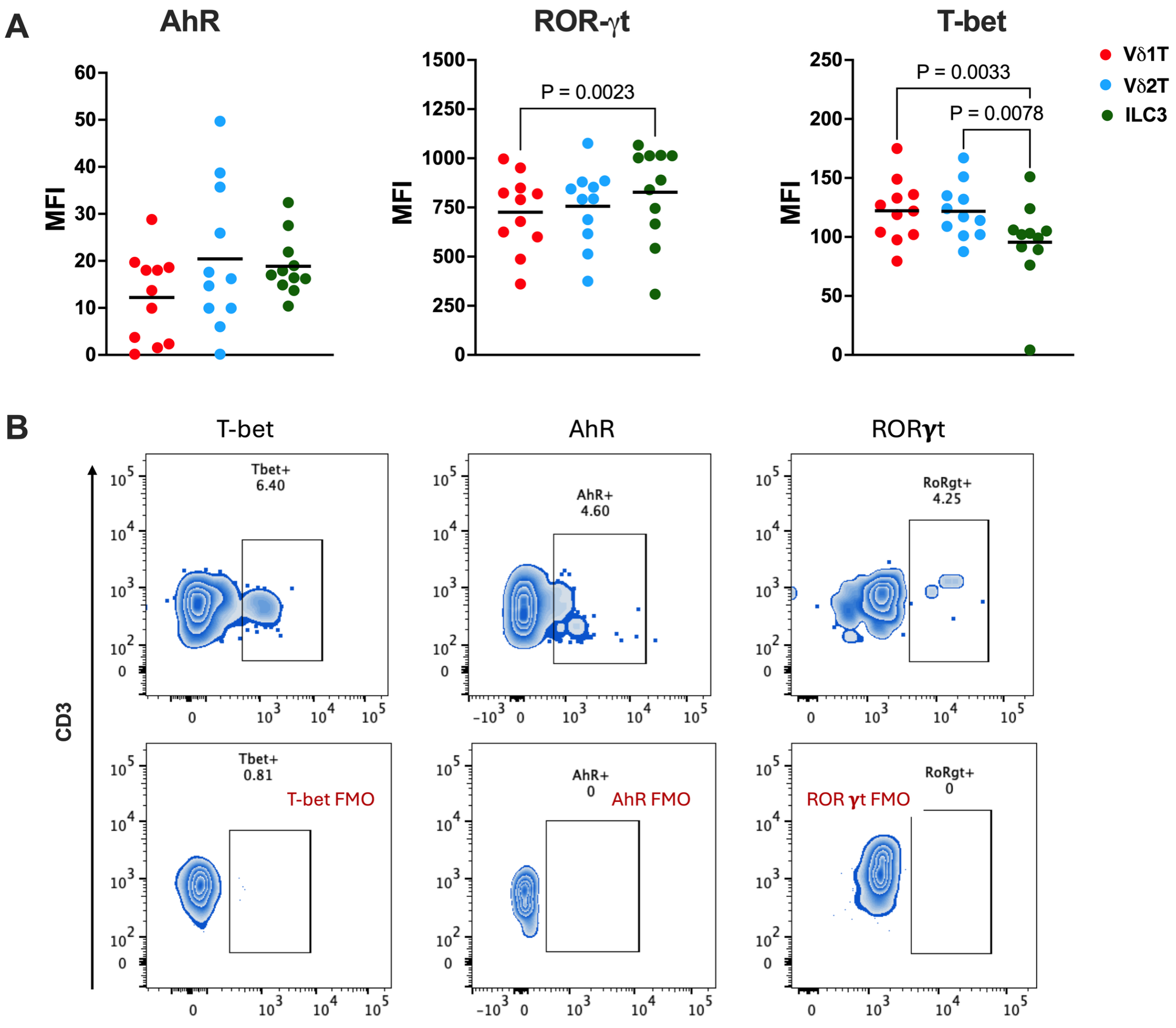

Supplemental Figure 2. CD4 and CD8 expression and memory phenotype of circulating Vδ1⁺ and Vδ2⁺ T cells. (A) Mean fluorescence intensity (MFI) of AhR, RORγt, and T-bet on Vδ1T cells (red symbols), Vδ2T cells (blue symbols), and ILC3 populations (green symbols) in PBMC. Data show Mean and P values for each comparison using one-way ANOVA. **(B)** Representative zebra plots of Vδ1 T cells showing Transcription factor staining (top panel) and respective FMO controls (bottom panel).

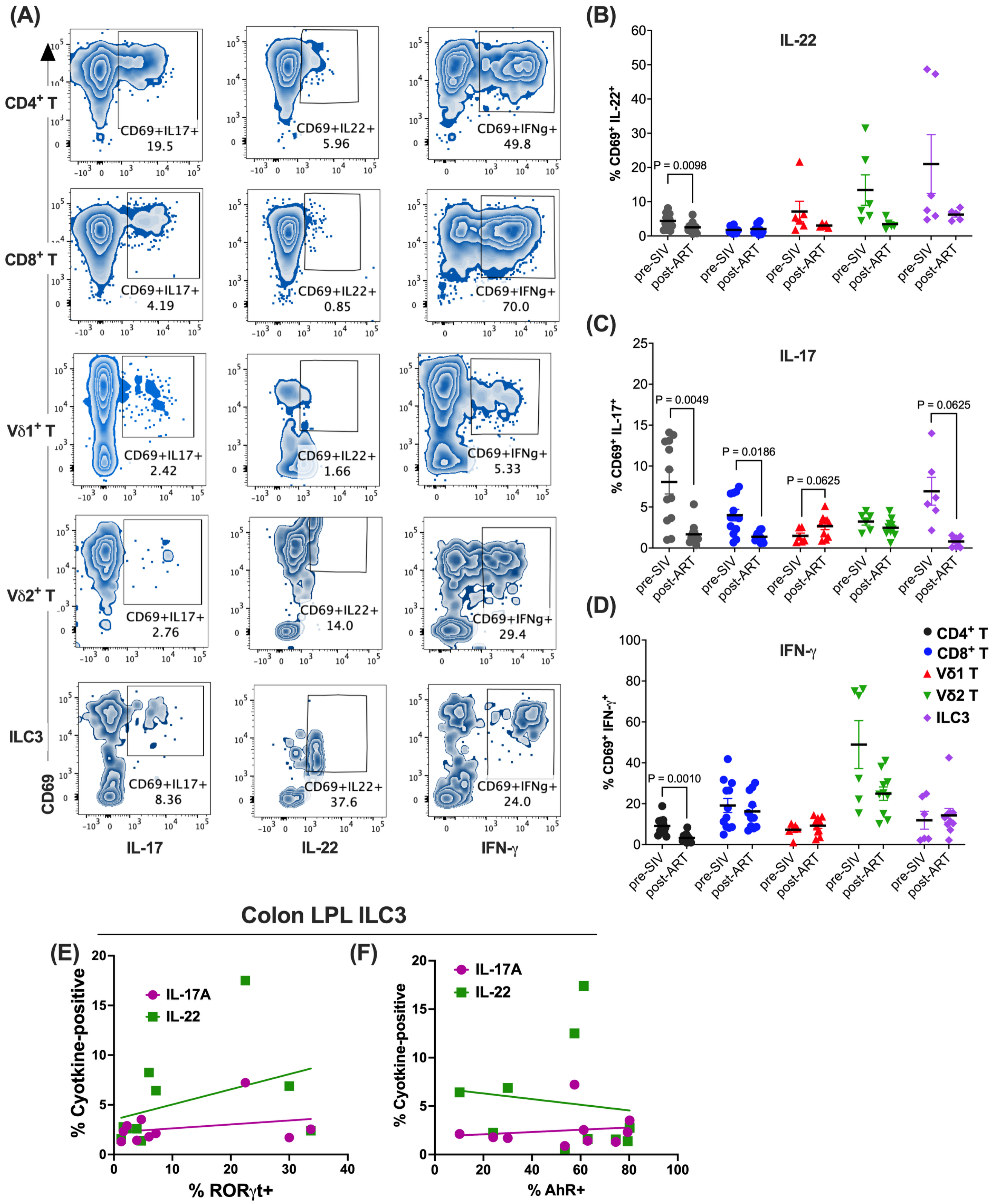

Supplemental Figure 3. Cytokine production by peripheral T cells and ILC3 during ART-suppressed SIV infection and correlation of colonic LPL ILC3 cytokines with transcription factor expression. Representative flow cytometry plots **(A)** and frequencies of CD4+ T cells, CD8+ T cells, γδ T cells, and ILC3s expressing IL-22 **(B)**, IL-17A **(C)**, and IFN-γ **(D)** determined by ICS. Data represent mean ± SEM and Wilcoxon test P values. Spearman correlation analysis of RORγt **(E)**, and AhR **(F)** expression with IL-17A and IL-22 cytokines produced by colonic ILC3 populations.

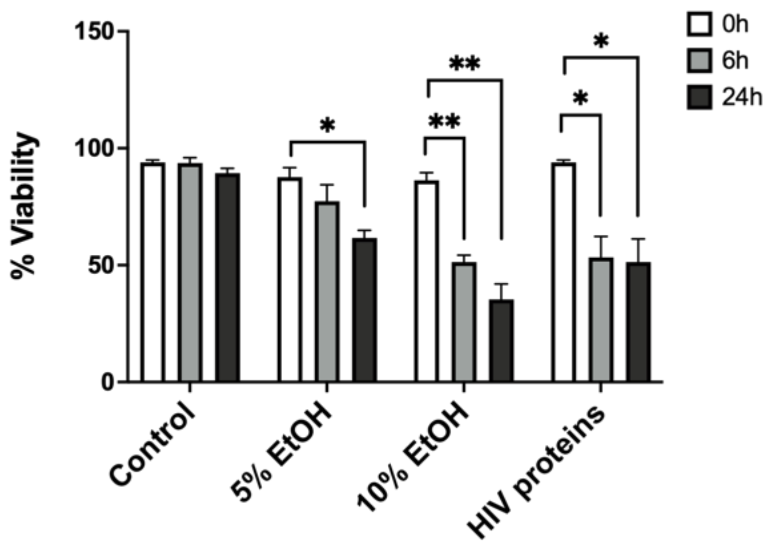

Supplemental Figure 4. Temporal effects of HIV proteins and ethanol on CaCo-2 cell viability. Caco-2 cell viability after the treatment with 5% EtOH, 10%EtOH, or HIV TAT+Nef+gp120 at 1μg/mL concentration. Viability was measured by trypan blue exclusion of dead cells at the indicated time-points. Paired t-test comparisons with matched wells at 0h prior to adding the treatments (*p<0.05, **p<0.01). Caco-2 cells alone served as negative controls at the indicated time-points.

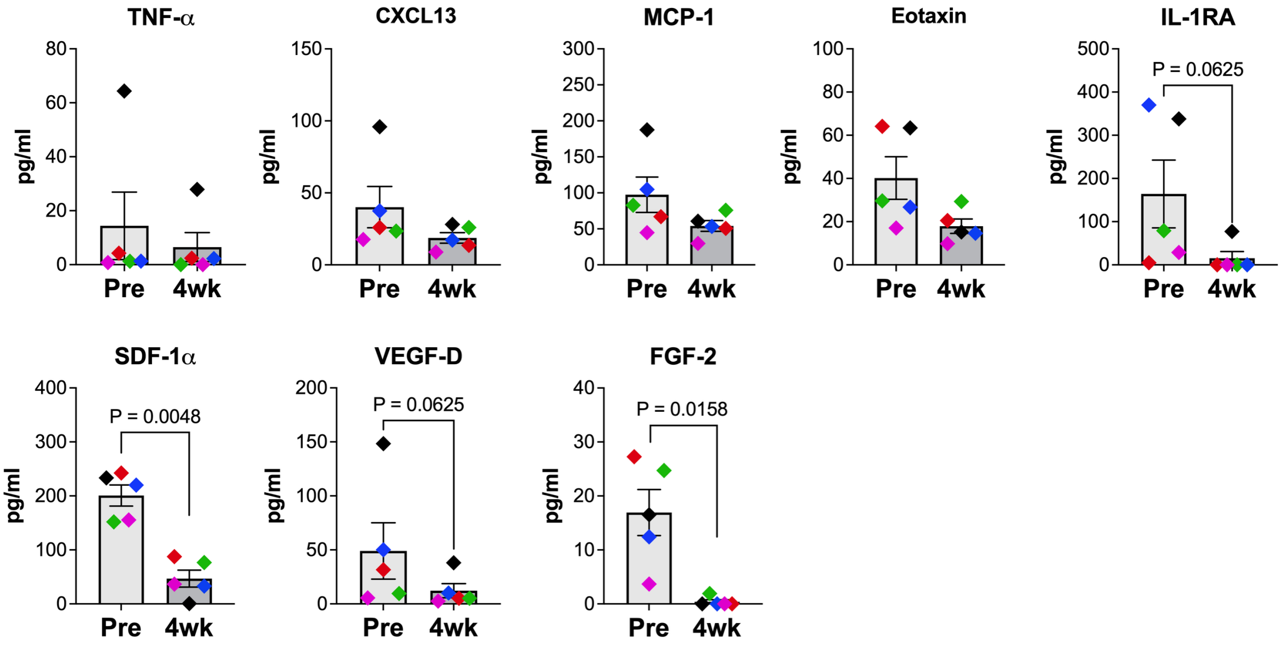

	MCP-1 Pre	MCP-1 4wk	EOTAXIN Pre	EOTAXIN 4wk	IL-1RA Pre	IL-1RA 4wk	SDF-a Pre	SDF-a 4wk	VEGF-D Pre	VEGF-D 4wk	FGF-2 Pre	FGF-2 4wk
**Test for normal distribution**												
**Shapiro-Wilk test**												
**W**	0.8951	0.974	0.8298	0.9386	0.8154	0.5522	0.819	0.9368	0.8045	0.7096	0.956	0.5522
**P value**	0.3834	0.9	0.1387	0.6557	0.1075	0.0001	0.115	0.6437	0.0882	0.012	0.7798	0.0001
**Passed normality test (alpha=0.05)?**	Yes	Yes	Yes	Yes	Yes	No	Yes	Yes	Yes	No	Yes	No
**P value summary**	ns	ns	ns	ns	ns	***	ns	ns	ns	*	ns	***
**Number of values**	5	5	5	5	5	5	5	5	5	5	5	5

Supplemental Figure 5. Effects of diet supplement on plasma levels of inflammatory cytokines and growth factors during SIV infection and ART. Plasma levels of the inflammatory cytokines, chemokines, and growth factors: TNF-α, CXCL13, MCP-1, Eotaxin, IL-1RA, Stromal Cell-Derived Factor 1 alpha (SDF-1α), Vascular Endothelial Growth Factor D (VEGF-D), and Fibroblast Growth Factor 2 (FGF-2), evaluated at 6 month-post-SIV+ART (pre-DS) and 4-weeks post-DS. P values shown for paired comparisons using t test for data that passed Shapiro-Wilk normality test (table below) and Wilcoxon test for analytes that were not normally distributed.

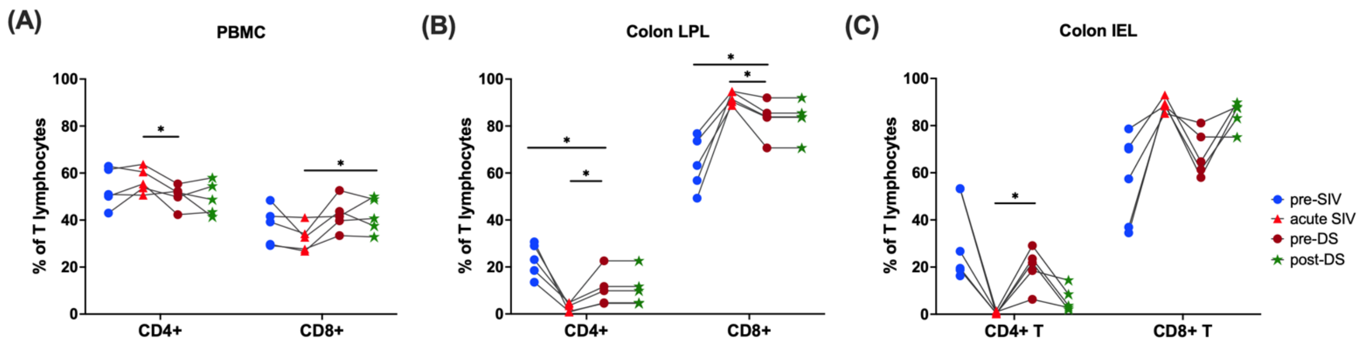

Supplemental Figure 6. Frequencies of classical T cells during chronic SIV+ART and following 4 weeks of broccoli-based diet supplementation. Ex vivo frequencies of $CD4^+$ T cells and $CD8^+$ T cells in **(A)** PBMC **(B)** colonic LPL, and **(C)** colonic IEL assessed longitudinally by flow cytometry. Data represent mean ± SEM in 5 animals: paired ANOVA (*$p < 0.05$).

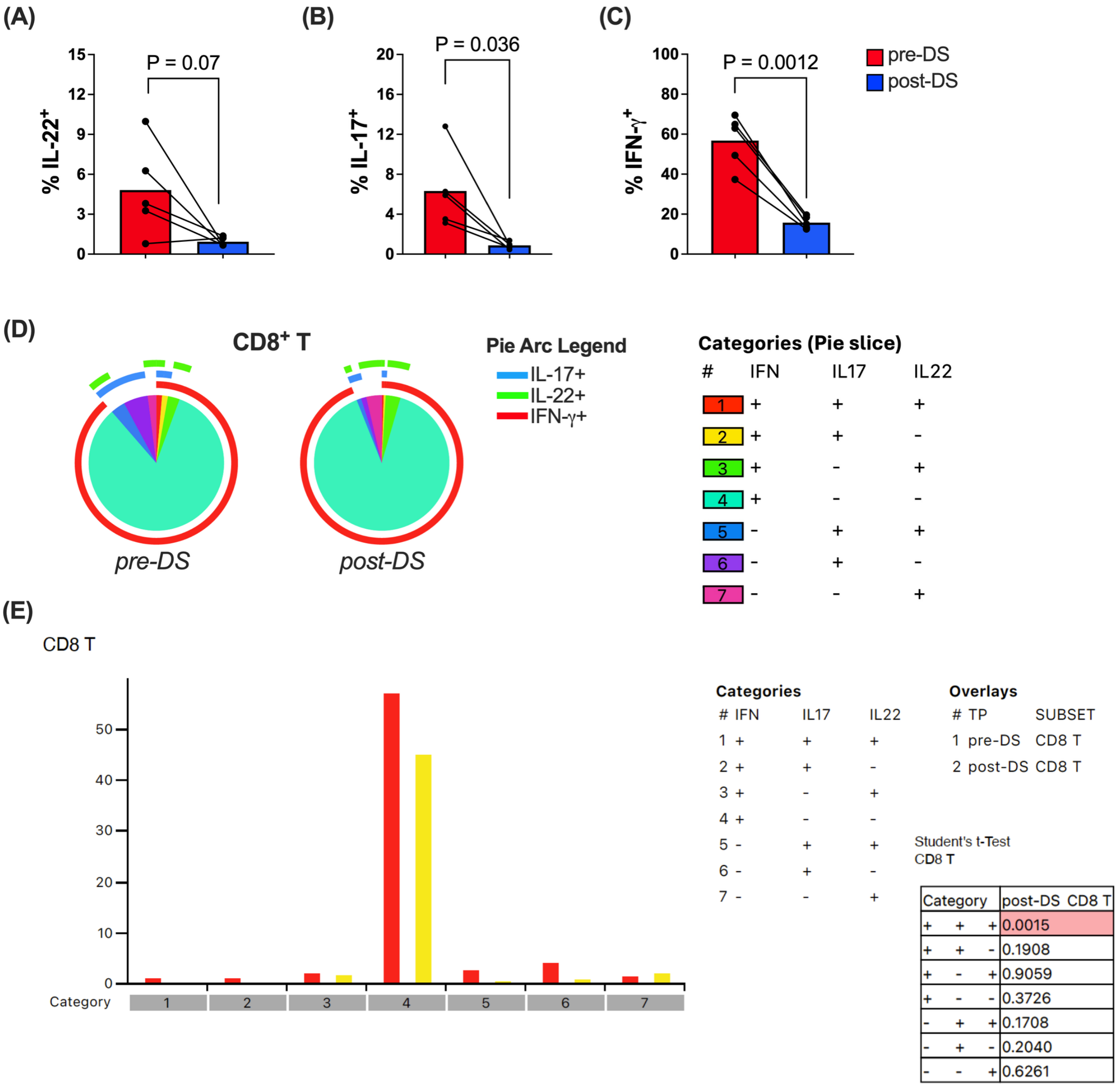

Supplemental Figure 7. Cytokine producing functions of CD8+ T cells in colonic LPL of rhesus macaques during chronic SIV+ART and following 4 weeks of broccoli-based diet supplementation. Intracellular cytokine staining for IL-22 **(A)**, IL-17A **(B)**, and IFN-γ **(C)** in CD8+ T cells following PMA/ionomycin stimulation of colonic LPL. Data represent mean ± SEM in 5 animals; paired ANOVA test. **(D)** Pie charts depicting changes in the intracellular expression of cytokines by CD8+ T cells in colonic LPL fraction from pre- and post-DS. **(E)** Differences in percentages of each combination of IFN-γ, IL17A, and IL-22 producing cells at pre-DS (red bar) and post-DS (yellow bar) time-points.

Supplemental Table 1. Phenotyping panel (Antibodies used for surface markers and transcription factor staining):

Marker	Clone	Company	Fluorochrome
TCR Vδ1	TS8.2	Invitrogen	FITC
RoRγt	AFKJS-9	Invitrogen	PCP-Cy5.5
T-bet	4B10	Biolegend	BV421
TCR γδ	B1	Biolegend	BV510
CD4	OKT4	Biolegend	BV605
CD8	SK1	BD	BV650
CD14	M5E3	BD	BV711
CD45	D058-1283	BD	BUV395
CD95	DX2	BD	BUV737
CD20	2H7	BD	BUV805
a4b7	A4B7R1	NHP Resource	PE
AhR	T49-550	BD	PE-CF594
CD28	CD28.2	BD	PE-Cy5
CD127	eBioRDR5	Invitrogen	PE-Cy5.5
TCR Vδ2	15D	Thermoscientific	APC
FVS700	----	BD	AL700
CD3	SP34-2	BD	APC-Cy7

Supplemental Table 2. Intracellular Cytokine Staining panel:

**Marker**	**Clone**	**Company**	**Fluorochrome**
TCR Vδ1	TS8.2	Invitrogen	FITC
IL-22	IL22JOP	Invitrogen	PCP-Cy5.5
CD4	OKT4	Biolegend	BV605
CD8	SK1	BD	BV650
CD14	M5E3	BD	BV711
CD45	D058-1283	BD	BUV395
PD-1	EH12.1	BD	BUV615
CD95	DX2	BD	BUV737
CD20	2H7	BD	BUV805
IL-17A	eBio64DEC17	Invitrogen	PE
CD69	FN50	BD	PE-CF594
CD161	DX12	BD	PE-Cy5
CD127	eBioRDR5	Invitrogen	PE-Cy5.5
IFN-γ	B27	BD	PE-Cy7
TCR Vδ2	15D	Thermoscientific	APC
FVS700	---	BD	AL700
CD3	SP34-2	BD	APC-Cy7

Supplemental Table 3. Antibodies and reagents for Multiplex Immunohistochemistry.

**Primary Antibody**	**Species**	**Clone**	**Company**	**Catalog**	**Dilution**
ZO-1	Mouse	ZO1-1A12	Invitrogen	33-9100	1:1000
Cytokeratin (AE1/AE3)	Mouse	AE1/AE3	Dako	M3515	1:400
AhR	Rabbit	polyclonal	GeneTech	GTX129013	1:1000
RORγt	Mouse IgG2a	6F3.1	Biocare Medical	ACI3208A	1:100
DAPI	–	–	Invitrogen	D1306	1:20,000

Supplemental Table 4. Description of study animal characteristics.

Animal ID	Group	Sex	Age (years)	Weight (kg)	Peak Viral Load (copies/mL)
RM1	DS	F	8.79	6.14	4.6
RM2	DS	M	5.81	10.34	7.4
RM3	DS	F	5.45	5.47	7.8
RM4	DS	M	4.69	10.47	7.8
RM5	DS	M	4.57	7.91	7.6
RM6	Control	F	10.22	9.65	8.2
RM7	Control	F	6.35	6.5	7.2
RM8	Control	F	5.98	5.85	7
RM9	Control	F	6.28	6.55	7.4
RM10	Control	F	5.37	8.4	6.6
RM11	Control	F	5.15	5.9	7.2
